# Competency Goals in Midwifery Master’s Programs in Germany and Selected OECD Countries: Comparison of Stakeholder Perspectives

**DOI:** 10.3390/healthcare14101377

**Published:** 2026-05-18

**Authors:** Angela Kranz, Anja Alexandra Schulz, Harald Abele, Joachim Graf

**Affiliations:** 1 Section of Midwifery Science, Institute of Health Sciences, University of Tübingen, 72074 Tübingen, Germany; harald.abele@med.uni-tuebingen.de (H.A.); joachim.graf@med.uni-tuebingen.de (J.G.); 2 Research Methods in the Health Sciences, University of Education Freiburg, 79117 Freiburg, Germany; anja.schulz@ph-freiburg.de; 3 Department for Women’s Health, University Hospital Tübingen, 72076 Tübingen, Germany

**Keywords:** advanced midwifery practice, competency-based education, midwifery educators, midwifery education, midwifery Master’s programs, stakeholder perspectives

## Abstract

Background: The increasing complexity of midwifery practice highlights the need for clearly defined competency goals in Master’s programs. Although international frameworks describe competency domains at the Master’s level, evidence on stakeholder prioritization remains limited, warranting an exploratory investigation. This international cross-sectional study examined stakeholders’ perceptions of the relevance of competency goals in midwifery Master’s programs across 19 selected OECD countries, with a predominance of respondents from Germany. Methods: N = 120 stakeholders rated 19 competency facets across three domains (general, advanced midwifery practice, and midwifery educator competencies) using a psychometrically validated 60-item questionnaire. A mixed-design MANOVA examined effects of profession (midwives vs. related professions), function (educators vs. applying professionals), and competency facets. Results: A significant within-subject effect across competency facets was identified (Pillai’s Trace = 0.492, *p* < 0.001). No significant main effects for profession (Pillai’s Trace = 0.012, *p* = 0.239) or function (Pillai’s Trace = 0.008, *p* = 0.325) were found, nor were significant interaction effects (Pillai’s Trace = 0.174, *p* = 0.311) observed. Conclusions: Given the exploratory design and the predominance of German participants, these findings provide a basis for future discussion on competency frameworks for midwifery Master’s programs. Clearly defined competency goals support the midwifery professionalization, quality of practice and the integration of advanced roles into complex care settings.

## 1. Introduction

The demands on midwifery practice and the associated competencies are becoming increasingly complex due to profound international changes in healthcare [1,2,3]. These include an increasing number of high-risk pregnancies [1,4], the impact of social developments such as poverty [1] and migration [5], health problems during pregnancy (e.g., multimorbidity [6] or chronic diseases [7]), and advancements in medical technology [8]. These developments require evidence-based care [3]. Legal developments at the international, European and national levels have contributed to the ongoing development of midwifery Bachelor’s and Master’s programs [9,10,11,12,13,14,15]. While Bachelor’s programs equip midwives with the competencies they need for basic professional qualifications and enable a rapid response to the global shortage of midwives [16], this does not equally apply to midwifery work in the demanding areas referenced above [17].

Midwifery Master’s programs can be distinguished into two types: post-nursing Master’s programs that serve as the primary qualification for registered nurses entering midwifery practice, and postgraduate Master’s programs that build on an existing midwifery Bachelor’s qualification [16]. The present study focuses on postgraduate Master’s programs that extend basic midwifery competencies with advanced academic skills [18].

The overarching objective of midwifery Master’s programs is to enable graduates to act effectively across professional levels [19]. Graduates provide advanced clinical care [20], contribute to the improvement in health systems through leadership competencies [21,22], foster interprofessional collaboration [23], generate new knowledge by conducting and applying research [24] and educate the future generation of midwives [25].

Competency-based curricula in midwifery Master’s programs require clearly defined competency goals in order to assess practical skills, taking into account the professional requirements of maternal and neonatal care [19,26]. Competency goals describe the expected knowledge, skills, attitudes and professional behaviors of students to cope with complex professional requirements and serve as learning outcomes in the context of competency-based education. Competency goals guide curriculum development, as well as the teaching strategies and assessment methods [19,26]. Beyond their educational function, clearly defined competency goals provide a shared reference framework that guides clinical practice [27,28], supports professional role development [29] and enables the structured integration of advanced midwifery roles into healthcare settings [30]. In this way, they contribute to both the professionalization of midwifery and the quality of midwifery care [2].

The following sections present the competency domains of midwifery Master’s programs (Section 1.1), the role of different stakeholder groups in shaping competency goals (Section 1.2) and the rationale of the study, as well as the hypotheses (Section 1.3).

### 1.1. Relevant Competency Domains in Midwifery Master’s Programs

Previous research by Kranz et al. [19,31] identified three overarching competency domains central to midwifery Master’s programs, operationalized by several competency goals: (1) general competencies in midwifery Master’s programs [27]; (2) specific competencies for Advanced Midwifery Practice (AMP) [30] and (3) specific competencies for midwifery educators [25]. These competency domains reflect the professional levels and roles midwives fulfill in increasingly complex healthcare settings [19,31]. Their systematic derivation from international competency frameworks is reported elsewhere [19]. The operationalization of the three competency domains [25,27,30] into specific frameworks adopted in the survey instrument is detailed in the accompanying validation study [31].

The domain “general competencies” focuses on foundational yet advanced competencies [27], such as research and evidence-based practice [32], best-practice models [33], midwifery theories [34], professional responsibilities and interprofessional collaboration [35,36], finance and management [21] and the use of technology [37]. This competency domain enables graduates to integrate academic knowledge into clinical practice [27].

The second competency domain encompasses competencies for AMP, representing an advanced level of midwifery expertise [30]. Building on the academic and research competencies of the first domain [27], the competencies of AMP emphasize advanced clinical decision-making, leadership skills [30,38] and interdisciplinary quality and risk management [30,39]. AMP is characterized by the delivery of individualized, evidence-based care in complex clinical situations [38,40]. Competencies for AMP reflect the evolving scope of midwifery practice at an advanced level, allowing midwives to shape and influence the midwifery profession and improve outcomes [30,39].

The third competency domain addresses competencies for midwifery educators, extending the general academic competencies with didactical and pedagogical expertise and thereby preparing the next generation of midwives [25]. This includes competencies such as didactics [41], quality assurance in educational programs [3] and research culture in teaching [42]. Midwifery educators are described in the literature as central actors for the sustainability, quality and further development of midwifery practice [25].

Given the partial overlap of the competency domains and the diverse needs of healthcare settings, the general competencies are designed to be implemented alongside one specialized domain (AMP or midwifery educator) [31,43].

To assess stakeholders’ relevance ratings of competencies in midwifery Master’s programs, the psychometrically validated survey instrument developed by Kranz et al. [19,31] was used. The instrument encompasses 60 items, structured into three competency domains [25,27,30] and divided into 19 competency facets (see Table 1). The multidimensional structure of the instrument was confirmed in previous research [31]. These empirically derived factors serve as the dependent variables for the hypotheses testing in the present study.

Despite the increasing relevance of midwifery Master’s programs [25,27,30], there is currently no internationally harmonized agreement on the competencies of these programs, reflecting diverse healthcare systems, varying educational pathways and differing stakeholder and political priorities at the national and international levels [18]. This lack of consensus results in international variations in program structures and competency goals [19].

### 1.2. Stakeholders’ Involvement in Midwifery Master’s Programs Curricula

Given the well-documented gap between academic curricula and healthcare realities [44], structured collaboration between education and health sector stakeholders has been recommended [45]. Involving stakeholders from academia, clinical practice and students as the next generation of midwives has been associated with greater clinical relevance of competencies, shared ownership and professional identity among students and curricula that better reflect the practical realities of midwifery practice [46,47,48]. Participatory curriculum processes have been linked to improvements in program quality and innovation in response to healthcare developments [49].

The perspectives of stakeholders in midwifery Master’s programs on competency goals derive from their different prioritizations, influenced by their background, professional focus and practical experience [49]. The comparison between midwives and related professions (e.g., physicians, nurses and other health professions) in their assessment of competency goals is central, reflecting interprofessional midwifery education and practice [2], as they are closely intertwined [50]. This is central in specialized areas requiring a Master’s degree, such as AMP [39,51]. Midwives, as an emerging academic profession, may place particular importance on competencies that strengthen professional autonomy, evidence-based practice and decision-making, as well as quality assurance. While these competencies are also integral to the medical practice of other healthcare professionals, such as physicians [52,53], for midwives these areas also serve as a mechanism for professional legitimation and autonomy within interprofessional healthcare systems [54,55]. This includes the competency areas of evidence-based practice (general competencies, factor 1) [39], midwifery research (general competencies, factor 2) [32], the analysis of health policy (general competencies, factor 3) [43], the identification of midwifery theories (general competencies, factor 5) [28], the professional understanding of the midwifery profession in different contexts (general competencies, factor 8) [27], and the promotion of quality care (AMP competencies, factor 3) [28]. Related professions, shaped by their own disciplinary background [54], are expected to potentially prioritize competencies central for clinical work in interprofessional healthcare teams [23], such as interprofessional collaboration and communication (general competencies, factor 4) [56], the use of technologies (general competencies, factor 6) [57] and the management of finances and resources (general competencies, factor 7) [58].

Comparing the stakeholder group midwifery educators with applying professionals addresses the gap between academic teaching and the practical application of competencies in clinical practice [49,59].

Midwifery educators (academic midwives and related professions [25]) are expected to prioritize competency facets related to didactics and curriculum development due to their pedagogical expertise and curricular responsibilities [25,60,61], including creating a conducive learning environment in theory and practice (midwifery educator competencies, factor 3–4) [25], quality assurance in educational programs (midwifery educator competencies, factor 5) [62] and incorporating research culture into teaching (midwifery educator competencies, factor 7) [63].

Practitioners, including related professions and midwives in practice, are characterized by a practical, application-oriented perspective in everyday professional life [64]. Students enrolled in midwifery Master’s programs represent a particularly relevant stakeholder group, as they are the direct recipients of competency-based education [65] and, in many contexts, simultaneously active practitioners in clinical settings [19]. They are therefore expected to assess competency goals from a practice-oriented standpoint aligned with practitioners [66,67]. On this basis, both groups were combined as “applying professionals” (practitioners and midwifery Master’s students), emphasizing competencies directly supporting clinical practice and theory–practice transfer [59].

This includes competencies to advocate and act as a leader (midwifery educator competencies, factor 6) [25], in leadership and accountability (AMP competencies, factor 2) [68] and in the ability to maintain and update midwifery competencies in theory and practice (midwifery educator competencies, factor 2) [69], as well as collaborative risk management competencies (AMP competencies, factor 4) [39].

While differences across these groups can occur due to their varied roles, consensus regarding the general principles and values for midwifery practice is expected, including ethical and legal principles in midwifery (midwifery educator competencies, factor 1) [25,70] as well as respect, dignity, trust and discretion (AMP competencies, factor 1) [28,71].

### 1.3. Rationale of the Study

While the international definition of competency goals for midwifery Master’s programs has become increasingly relevant in recent years [25,27,30], empirical research examining how different stakeholders assess the relevance of these competencies remains limited. It is unclear whether consensus or divergence exists among the stakeholder perceptions of competency goals. Considering their perspectives supports the development of applicable, pedagogically sound and practice-oriented competency goals [47,72,73], contributing to discussion on the development of an evidence-based international curriculum for midwifery Master’s programs [19].

To address this research gap, this international cross-sectional study examined stakeholders’ assessments of competency goals in midwifery Master’s programs across selected Organization for Economic Cooperation and Development (OECD) countries. Given the predominance of German participants, the study is understood as exploratory, context-sensitive investigation rather than a representative international survey. Using a psychometrically validated questionnaire [31], this study assessed the perceived relevance of three main competency domains [25,27,30]. The hypotheses were derived from the existing literature and served to structure the statistical analysis. However, given the absence of established evidence for stakeholder comparisons in this specific field and the heterogeneity of the targeted population, the hypotheses reflect informed expectations rather than strong predictions. The hypotheses are structured according to the mixed MANOVA design. H1, H2 and H4 correspond to the three test levels of the analysis (main effect profession, main effect function, profession × function × competency facet interaction). The directional sub-hypotheses (H1a/H1b, H2a/H2b) specify which stakeholder group is expected to rate which competency facets higher. H3 addresses an expectation of no difference regarding competency facets that reflect principles and values shared across stakeholder groups.

**Hypothesis** **1.** *Midwives and related professions differ significantly in their combined assessment of the relevance of competency facets in midwifery Master’s programs (main effect profession)*.

**Hypothesis** **1a.** *Midwives rate competency facets related to evidence-based, independent midwifery practice and midwifery professionalization significantly higher in their relevance than related professions (general competencies, factors 1–3, 5, 8; AMP competencies, factor 3)*.

**Hypothesis** **1b.** *Related professions rate competency facets central to interprofessional collaboration, technology use and resource management significantly higher than midwives (general competencies, factors 4, 6, 7)*.

**Hypothesis** **2.** *Midwifery educators and applying professionals differ significantly in their combined assessments of the relevance of competency facets in midwifery Master’s programs (main effect function)*.

**Hypothesis** **2a.** *Midwifery educators rate didactic, curriculum-related competency facets significantly higher in their relevance than applying professionals (midwifery educator competencies, factors 3–5, 7)*.

**Hypothesis** **2b.** *Applying professionals rate competency facets directly supporting clinical practice and theory-practice transfer significantly higher in their relevance than midwifery educators (AMP competencies, factors 2, 4; midwifery educator competencies, factor 2, 6)*.

**Hypothesis** **3.** *All stakeholder groups do not differ significantly in their assessment of the relevance of competency facets related to fundamental principles and values shared across groups (midwifery educator competencies, factor 1; AMP competencies, factor 1)*.

**Hypothesis** **4.** *The extent and direction of group differences in the assessment of the relevance of competency facet ratings depend on the interaction between profession (midwives vs. related professions) and function (educators vs. applying professionals). The combined effect of profession and function varies across different competency facets (interaction effect profession x function x competency facet)*.

## 2. Materials and Methods

This international cross-sectional study used a psychometrically validated questionnaire [31] to survey stakeholders’ assessments of competency goals in midwifery Master’s programs in selected OECD countries, with a predominance of participants from Germany.

The ethical review was conducted by the Ethics Committee of the Medical Faculty of the University Hospital and the University of Tübingen (review number: 404/2023B02, date: 27 July 2023). All requirements of the General Data Protection Regulation (GDPR) were met, including active and informed consent to participate in the study (see Supplementary Materials S1 and S2) [74].

### 2.1. Sample: Inclusion Criteria, Recruitment and Group Classification

Participants included (1) educators in midwifery Master’s programs, (2) educators in midwifery Bachelor’s programs, (3) students in midwifery Master’s programs and (4) practitioners collaborating with academic midwives. To ensure comparability across health and education systems [43,75]—and because most midwifery Master’s programs are located in these countries [3,16]—participants were required to work or study within an OECD country. Additionally, participants had to be at least 18 years old, able to provide informed consent and be sufficiently proficient in German or English to complete the questionnaire. Due to the absence of an international consensus on relevant stakeholders for midwifery Master’s programs [44], the study adopted an exploratory approach and did not aim for statistical representativeness of all OECD countries.

Between February and May 2024, a non-probability sample was recruited using the self-selection, quota and snowball sampling methods. A public call for participation was disseminated through 38 international midwifery associations. In 21 OECD countries, educators in midwifery Master’s programs (*n* = 485), educators in midwifery Bachelor’s programs (*n* = 275) and leading midwives and related professions in obstetrics departments (*n* = 154) were contacted via publicly available email addresses. The recruitment letter (see Supplementary Materials S1 and S2) encouraged participants to share the survey invitation with their professional networks, especially with students in midwifery Master’s programs, as no public contact information was available for this group. Additionally, approximately 100 potential participants were approached during two national conferences in Germany. In total, complete data from N = 120 individuals could be included in the data analysis.

### 2.2. Sample Size and Power

To determine a significant population effect, an a priori power analysis for the mixed MANOVA was conducted using the software tool G*Power (version 3.1) (in G*Power: ANOVA: repeated measures, within–between interaction) [76]. In line with conventions in educational research, where a small-to-medium effect size of Cohen’s d ≈ 0.40 is typically considered practically relevant, the present study assumed a corresponding small-to-medium effect size of Cohen’s f = 0.20 [77,78]. With a test power of 1–β = 0.80, a significance level of α = 0.05, number of measurements = 19 (one per competency facet), correlation among repeated measures r = 0.3 and ε = 0.75, the power analysis resulted in a total required sample size of N = 40. Given the empirically identified discriminant validity among the competency facets [31], the average intercorrelation among repeated measures was expected to be low to moderate, supporting the assumption of r = 0.30 [79]. Similarly, ε = 0.75 was selected to account for potential moderate violations of sphericity, which are likely with a larger number of repeated measurements [79].

To increase the statistical power, the initially defined participants were consolidated into the following overarching groups:

- Midwives (*n* = 89) vs. related professions (*n* = 31).
- Educators (Bachelor’s and Master’s programs) (*n* = 66) vs. applying professionals (Master’s students and practitioners) (*n* = 54).

### 2.3. Survey Instrument

The survey instrument (see Supplementary Materials S3 and S4) assessed the perceived relevance of competency goals in midwifery Master’s programs across selected OECD countries. It comprised 60 items in both German and English via the online platform LimeSurvey. After 10 sociodemographic variables, the items for the three competency domains identified by Kranz et al. [19] were as follows: (1) general competencies in midwifery Master’s programs (30 items); (2) specific competencies for AMP (9 items); (3) specific competencies for midwifery educator (21 items). Based on international competency frameworks [25,27,30], the original competency statements were inductively operationalized into items and grouped into competency facets/factors (see Supplementary Materials S3 and S4). Each item represented a specific competency statement, beginning with “Ability to…” and was rated on a five-point unipolar rating scale [80] (“1” = Not important in the Master’s program to “5” = Very important in the Master’s program). Participants were asked the following question: When students start the Master’s program, they already have acquired basic competencies of midwifery practice. To what extent is it important that the following competencies are developed or deepened in the Master’s program? Using the same structure, four control items at the Bachelor’s level were added, based on the International Confederation of Midwives (ICM) standards [12], to assess the distinction between Bachelor’s and Master’s competencies.

The German version was prepared by bilingual members of the research team who were native speakers of both languages, working from the English-language source materials of the underlying frameworks. The pretest (reported in [31]) included both German- and English-speaking participants and led to linguistic refinements in both versions. However, a formal cross-cultural adaptation following international guidelines [81] was not conducted (see Section 4.2, Limitations).

The operationalization of the three competency domains with the corresponding competency facets/factors was tested for factorial validity and reliability in prior work using a CFA and reliability analysis [31]. The results indicated a multidimensional structure of these domains, with an eight-factorial solution for general competencies, a four-factorial solution for AMP competencies and a seven-factorial solution for midwifery educator competencies, each demonstrating acceptable to good model fits. Reliability (Cronbach’s Alpha) was acceptable to good for all identified competency facets (general competencies: α = 0.798–0.946; AMP competencies: α = 0.797–0.960; educator competencies: α = 0.782–0.975) [31].

### 2.4. Mixed Multivariate Analysis of Variance (MANOVA)

To test the hypotheses outlined above, a mixed-design multivariate analysis of variance (MANOVA) was conducted. Competency facets were the multiple dependent variables modeled as repeated measures as within-subject factors (each participant provided ratings across all competency facets, creating correlated repeated measurements within individuals), and the stakeholders’ profession (midwives vs. related professions) and function (midwifery educators vs. applying professionals) served as the between-subject factors. The mixed MANOVA design allowed for the examination of main effects for profession and function as well as their interaction effects with 19 repeated measures (competency facets) [79,82]. Despite the separate theoretical derivation and analysis of the three competency constructs [31], their intercorrelations were examined by correlating the mean scores of each construct. The construct-level intercorrelations (Pearson’s r) indicated moderate to strong relationships (r = 0.463–0.671, all *p* < 0.001; see Appendix A Table A1). In line with recommendations for multivariate analysis that correlated outcome domains should be analyzed jointly rather than in separate univariate models [79], all 19 competency facets of the three constructs were analyzed in a single mixed MANOVA.

The statistical assumptions for the mixed MANOVA were tested. The multivariate normality of residuals and their independence within each profession-function cell were tested using skewness and kurtosis for all competency facets and by screening for univariate and multivariate outliers (Q-Q plots and Mahalanobis distances) [79,82]. Multivariate outliers, using Mahalanobis distances, were examined via linear regression with all 19 competency facets as predictor variables and a dependent variable for technical purpose only (not interpreted). Each Mahalanobis distance was compared against a chi-square distribution with 19 degrees of freedom [82]. As the within-subject factor comprised multiple repeated measures (19 measures), the assumption of sphericity was evaluated using the Mauchly’s sphericity test. Greenhouse–Geisser ε-corrections were applied as needed to adjust degrees of freedom when sphericity was violated (*p* < 0.05) [79]. With product–moment correlation matrices, linear relationships between the dependent variables were examined (0.10 = small; 0.30 = medium; 0.50 = large), while ensuring the absence of problematic multicollinearity (very high intercorrelations, no correlations exceeded r ≈ 0.85) [79,82]. The homogeneity of the variance-covariance matrices across the four stakeholder groups was evaluated with the Box’s M test [79,82]. Levene’s test was used to assess the quality of error variances for each dependent variable [79,82]. Adequate cell sizes are generally required to ensure stable estimations of covariance matrices [82]; in the present study, this assumption was only partly met. Pillai’s trace was therefore used as the primary multivariate test statistic because it is comparably robust to moderate violations of these assumptions, especially unequal cell sizes [79,82]. Effect sizes for multivariate effects were reported using partial eta η^2^_p_, with values of 0.01 (small effect), 0.06 (medium effect) and 0.14 (large effect) [79]. The significance level was set at *p* < 0.05.

The estimated marginal means (EMMEANS) derived from the general linear model were calculated to test the a priori-defined and theory-driven contrasts (see Hypotheses 1a, 1b; 2a, 2b) [83]. Bonferroni adjustment was applied to control the familywise error rate for multiple planned comparisons, whereas adjusted and unadjusted *p*-values are identical for single pairwise comparisons [79].

All statistical analyses were calculated using IBM SPSS Statistics version 31.0.1.

## 3. Results

### 3.1. Socio-Demographic Characteristics and Descriptive Statistics

The total sample included N = 120 participants. Regarding their profession, *n* = 89 were midwives and *n* = 31 were in a related profession. In terms of their function, *n* = 66 participants were educators in midwifery Bachelor’s or Master’s programs and *n* = 54 applying professionals. Additional socio-demographic characteristics of the sample were previously described and discussed by Kranz et al. [31], based on the same dataset. Details are provided in the Appendix A Table A2. Descriptive statistics at the item level (see Appendix A Table A3) revealed that the general competencies (construct I) were rated as having the highest scores across all stakeholder groups, with item means ranging between $x^{-}$ = 3.85 (I-3-1, health policy analysis) and $x^{-}$ = 4.56 (I-1-3 evidence-based decision making) on the 1–5 scale. Within this construct, items reflecting evidence-based practice reached means above $x^{-}$ > 4.33 (e.g., I-1-1 $x^{-}$ = 4.33 (identify gaps between evidence and practice); I-1-2 $x^{-}$ = 4.46 (bridging gaps between evidence and practice); I-1-3 $x^{-}$ = 4.56 (evidence-based decision making); I-1-4 $x^{-}$ = 4.43 (promoting evidence-based midwifery practice); I-1-5 $x^{-}$ = 4.42 (integrate research findings into practice)). Items in construct II (AMP) were rated slightly lower overall, with item means ranging between $x^{-}$ = 3.45 (II-3-2, promote and protect compassionate maternity care and practice) and $x^{-}$ = 4.21 (II-2-2, advanced knowledge and skills within a leadership role). Items relating to leadership and responsibility exceeded the highest item means for this construct (II-2-2 $x^{-}$ = 4.21 (advanced knowledge and skills within a leadership role), II-2-1 $x^{-}$ = 4.13 (advanced knowledge and skills in a leadership role in complex decision making)). In construct III (midwifery educator), item means were generally the lowest across all constructs, ranging between $x^{-}$ = 3.53 (III-6-1 communication methods in different settings) and $x^{-}$ = 4.25 (III-7-2 supportive culture of evidence-based practice). Items relating to research culture in teaching received the highest ratings (III-7-1 $x^{-}$ = 4.18 (use research findings to inform teaching and practice); III-7-2 $x^{-}$ = 4.25 (supportive culture of evidence-based practice); III-7-3 $x^{-}$ = 4.23 (promote culture of critical inquiry). Finally, the control items representing Bachelor’s-level competencies demonstrated mean values above the scale midpoint ($x^{-}$ > 2.50), ranging from $x^{-}$ = 3.40 (C-2 adhere jurisdictional laws, regulatory requirements, and codes of conduct for midwifery practice) to $x^{-}$ = 3.57 (C-3 facilitate normal birth processes in institutional and community settings). This indicates perceived relevance at the Master’s level but limited discriminatory capacity between qualification levels.

Descriptive statistics on the factor-level (see Appendix A Table A4) demonstrated that all stakeholder groups rated the competency facets as important, with mean scores generally being $x^{-}$ > 3.5 on a 1–5 scale. For construct I (general competencies), midwives and midwifery educators tended to rate the assessed competency facets slightly higher, although differences were small in magnitude across facets (midwives ($x^{-}$ = 3.523–4.486), related professions ($x^{-}$ = 3.597–4.310), midwifery educators ($x^{-}$ = 3.585–4.571), related profession educators ($x^{-}$ = 3.620–4.344), midwives applying professionals ($x^{-}$ = 3.469–4.413), and related professions applying professionals ($x^{-}$ = 3.389–4.167)). The competency facets of construct II (AMP competencies) were marginally lower overall, with midwifery educators demonstrating the highest means ($x^{-}$ = 3.731–4.293). For construct III (midwifery educator competencies), midwifery educators consistently provided the highest mean scores ($x^{-}$ = 3.842–4.439), whereas applying professionals from related professions often demonstrated the lowest mean scores ($x^{-}$ = 2.611–4.389).

### 3.2. Mixed MANOVA: Testing of Assumptions

Shapiro–Wilk tests indicated significant deviations from normality for most of the competency facets across stakeholder groups (*p* < 0.001), with only a few exceptions (e.g., competency facet F1_F6 for related profession educators *p* = 0.053). Across competency facets and groups, skewness values were predominantly negative, indicating a concentration of higher importance ratings on the upper end of the scale (values ranged from F1_F1 for related profession educators −1.941 (SE = 0.464) to F1_F7 for related professions applying professionals 1.148 (SE = 0.845)). Kurtosis values ranged from approximately mesokurtic to moderately leptokurtic (values ranged from F2_F2 for related professions applying professionals −2.390 (SE = 1.741) to F1_F1 for related professions educators (6.069 (SE = 0.902)). None of the skewness values exceeded the critical threshold of |3| and all kurtosis values remained below |8| [79,84], supporting acceptable normality for parametric analyses despite the Shapiro–Wilk violations (see Appendix A Table A5). No univariate outliers were detected upon scanning the Q-Q plots. No cases exceeded the critical chi-square value of *p* < 0.001 when analyzing the Mahalanobis distances, indicating no problematic multivariate outliers occurred in the dataset [82]. The examination of linear relationships between the 19 competency facets demonstrated small to large correlations (r = 0.011 (F1_F3 and F3_F7)–r = 0.669 (F3_F3 and F3_F4), supporting the linearity assumption (see Appendix A Table A6). No multicollinearity was evident (all correlations were below r = 0.85). Box’s M test indicated a significant violation of the assumption of equality of variance–covariance matrices (M = 608.63, F (380, 18,539.72) = 1.159, *p* = 0.019). Mauchly’s Test indicated a violation of sphericity for the within-subject factors (*p* < 0.001). Levene’s test confirmed the assumption of homogeneity of variances for most variables (*p* > 0.05), with one significant group difference in variances in the first construct (competency facet F1_F3, *p* = 0.041) (see Appendix A Table A5).

### 3.3. Mixed MANOVA: Testing of Hypotheses

Table 2 demonstrated a significant multivariate within-subject effect of competency facets, indicating differences in the assessed relevance ratings across the competency facets (Pillai’s Trace = 0.492, F (18,99) = 5.34, *p* < 0.001, η^2^_p_ = 0.492 (within-subject main effect)).

The multivariate main effect of profession was not significant, indicating that midwives and related professions did not differ significantly in their combined assessment of the relevance of competency facets in midwifery Master’s programs, rejecting Hypothesis 1 (Pillai’s Trace = 0.012; F (1,116) = 1.401; *p* = 0.239; η^2^_p_ = 0.012) (main effect profession).

The multivariate main effect of function was not significant, indicating that midwifery educators and applying professionals did not differ significantly in their combined assessment of the relevance of competency facets in midwifery Master’s programs, rejecting Hypothesis 2 (Pillai’s Trace = 0.008; F (1,116) = 0.975; *p* = 0.325; η^2^_p_ = 0.008) (main effect function).

The combined effect of profession and function did not vary across different competency facets, as the three-way interaction between competency facets, profession and function was not significant, rejecting Hypothesis 4 (Pillai’s Trace = 0.174; F (18,99) = 1.157; *p* = 0.311; η^2^_p_ = 0.174) (interaction effect profession x function x competency facet).

Hypothesis 1a was rejected. Although midwives rated all competency facets related to evidence-based, independent midwifery practice and midwifery professionalization (general competencies, factors 1–3, 5, 8; AMP competencies, factor 3) descriptively higher in their relevance than related professions, none of the a priori-defined contrasts were statistically significant (all *p* ≥ 0.144). Mean differences ranged from MD = 0.054 (F2_F3) to 0.227 (F1_F5), with all 95% confidence intervals including zero (see Table 3).

Hypothesis 1b was rejected. Related professions rated competency facets central to interprofessional collaboration, technology use and resource management (general competencies, factors 4, 6, 7) descriptively higher in their relevance than midwives across all relevant facets. However, none of the a priori-defined contrasts were statistically significant (all *p* ≥ 0.490). Mean differences ranged from MD = −0.074 (F1_F6) to −0.136 (F1_F4). The 95% confidence intervals were not significant, including zero (see Table 3).

Hypothesis 2a was rejected. Midwifery educators rated didactic, curriculum-related competency facets (midwifery educator competencies, factors 3–5, 7) descriptively higher in their relevance than applying professionals for three of the four relevant facets, whereas ratings were nearly identical for one facet (F3_F5). None of the differences were statistically significant (*p* ≥ 0.239). Mean differences ranged from MD −0.011 (F3_F5) to 0.240 (F3_F3) and all 95% confidence intervals included zero (see Table 4).

Hypothesis 2b was rejected. Although applying professionals rated competency facets directly supporting clinical practice and theory-practice transfer descriptively (AMP competencies, factors 2, 4; midwifery educator competencies, factor 2, 6) higher in their relevance than educators across all relevant facets, the differences were not statistically significant (*p* ≥ 0.214). Mean differences ranged from MD = 0.018 (F2_F4) to 0.193 (F2_F2), with all 95% confidence intervals including zero (see Table 4).

Hypothesis 3 was supported. No significant differences were identified between professions or functions in the assessment of the relevance of competency facets related to the fundamental principles and values shared across groups (midwifery educator competencies, factor 1; AMP competencies, factor 1), with mean differences ranging from −0.028 (F3_F1 function) to 0.286 (F3_F1 profession). All effects were non-significant with negligible effect sizes (all *p* ≥ 0.228, η^2^_p_ ≤ 0.012). All 95% confidence intervals included zero (see Table 5).

## 4. Discussion

In this cross-sectional study, a mixed MANOVA was conducted, which revealed no significant differences among profession or function in their assessment of the relevance of competency goals in midwifery Master’s programs—neither in the main effects nor in the interaction effects (profession: Pillai’s Trace = 0.012, F = 1.401, *p* = 0.239, η^2^_p_ = 0.012; function: Pillai’s Trace = 0.008, F = 0.975, *p* = 0.325, η^2^_p_ = 0.008; interaction: Pillai’s Trace = 0.174, F = 1.157, *p* = 0.311, η^2^_p_ = 0.174), despite a significant within-subject effect across competency facets (Pillai’s Trace = 0.492, F = 5.335, *p* < 0.001, η^2^_p_ = 0.492). Across all stakeholder groups, the assessed competency facets were consistently rated high, while none of the hypothesized group differences reached statistical significance at the multivariate or facet level. Overall, no statistically significant group-based differences were observed in the assessed relevance of competency goals in midwifery Master’s programs.

### 4.1. Stakeholders Perspectives on Competency Goals in Midwifery Master’s Programs

The present study examined stakeholders’ perceptions of the relevance of competency goals in midwifery Master’s programs across three competency domains: (1) general competencies in midwifery Master’s programs; (2) specific competencies for AMP and (3) specific competencies for midwifery educators.

At a descriptive level, all stakeholder groups rated general competencies in midwifery Master’s programs, especially evidence-based practice and research skills, with the highest mean scores. This pattern may reflect a broadly similar prioritization of core competencies, with general academic competencies as common reference point for advanced practice across roles and settings for midwives with a Master’s degree [19]. Evidence-based practice and research skills contribute to the core components of healthcare Master’s curricula, preparing graduates for analytically demanding roles in complex healthcare settings [32]. In contrast, competencies related to AMP and midwifery educators were rated slightly lower overall and demonstrated more differentiated descriptive patterns across stakeholder groups. These descriptive patterns suggest a distinction between foundational general competencies—perceived as universally essential—and role-specific specializations [19,85]. Specific competencies in the areas of AMP and midwifery educators can be offered as flexible specialization tailored to national regulations, institutional strengths and individual career paths, whereas core competencies, as described in the general academic competencies, remain central for every Master’s program in midwifery [19,27]. This distinction is particularly relevant given the limited time scope in a Master’s program, which is often tailored to four semesters [10]; this is why the implementation of all competency goals analyzed in this study appears challenging. This makes it central to define a limited set of core Master’s-level competencies. The general academic competencies identified in this study may serve as a starting point for future research about core competencies, complemented by context-specific specialization options. This overarching prioritization of general academic competencies is descriptively consistent with a broadly shared orientation towards analytical, evidence-based and research-oriented competencies as a central prerequisite for addressing the complex challenges in midwifery care, as described in the introduction [18,86].

The significant within-subject effect (Pillai’s Trace = 0.492; *p* < 0.001; η^2^_p_ = 0.492) indicates that ratings varied significantly across the 19 competency facets, which is consistent with the descriptive patterns observed. The findings show that the perceived relevance across competency domains are not rated as equally relevant, with general academic competencies receiving descriptively higher ratings than specialized domains. For the curriculum development, this pattern may provide a preliminary basis for prioritizing general academic competencies as a shared core goal, while AMP and midwifery educator competencies serve as context-specific specializations, as described above.

The non-significant multivariate main effects for profession (midwives vs. related professions) and function (midwifery educators vs. applying professionals), as well as non-significant interaction effects across competency goals, indicate that stakeholder perspectives on competency goals in midwifery Master’s programs do not derive from systematic group-based differences. These results suggest that professional identity and occupational function do not substantially influence the overall pattern of the perceived relevance for competencies in Master’s programs. These findings are in line with the literature indicating broad consensus among stakeholder perspectives on core competencies in health professions’ education, despite differing professional roles and functional positions [49,87]. Stakeholders often agree on relevant competencies, while their operationalization and application within specific institutional, regulatory and care contexts can differ [49,88]. The lack of statistically significant group differences could indicate that the academization and professionalization of midwifery has been broadly internalized across stakeholder groups [54]. At the same time, the small and unequal subgroup sizes limit the confidence with which these null results can be interpreted as evidence of absent group differences. The possibility of a Type II error cannot be excluded [79]. This concern applies in particular to the three-way interaction effect, whose detection is most strongly affected by the underpopulated subgroup.

The theoretically derived stakeholder contrasts did not translate into systematic differences in the statistical analysis of the competency goals. Clear a priori expectations suggested that stakeholder groups would prioritize competency facets differently. However, the mixed MANOVA did not yield statistically significant group differences. This is consistent with a shared understanding of the relevant Master’s competencies for midwives, although it does not provide evidence for it. In health profession education, this pattern is reported in the literature, where consensus among core competency goals across diverse stakeholder groups exists, even when they have differing educational and professional roles [49,89]. The absence of significant between-group and interaction effects does not negate the presence of meaningful differences among the competency goals themselves, which was demonstrated with the significant within-subject effect for competency facets. This suggests that differences are present between types of competencies, rather than within stakeholder group membership [47,79].

Overall, these findings show that the competency domains, competency facets and competency goals identified in previous work are not only psychometrically distinct [31], but also demonstrate no statistically detectable stakeholder-specific differences in the assessed relevance of advanced midwifery roles and competencies at the Master’s level across clinical and educational functions.

In addition to the methodological limitations discussed in the limitations section (Section 4.2), including reduced statistical power due to unequal subgroup sizes, ceiling effect, deviations from multivariate normality and the non-probability sampling strategy, several alternative interpretations of the absence of group differences should be considered. First, the high relevance ratings across all stakeholder groups may reflect a shared conception of midwifery Master’s competencies. This may have been internalized through professional socialization, international competency frameworks and curricular discourses [34,90], rather than the individual prioritization of Master’s competencies. Profession-specific values, norms and behaviors may lead to homogeneous response patterns [91]. Second, the abstract level at which competency facets were operationalized may have made it more difficult to detect nuanced differences in stakeholders’ perceptions [80]. Third, given that more than half of the participants were recruited from the German national context, with shared regulatory, curricular and healthcare system frameworks, this may have led to the homogeneity of ratings rather than role-specific orientations [43,92]. Fourth, the recruitment strategy may have led to self-selected samples of stakeholders engaged with the academization of midwifery [93]. Stakeholders that are critical of midwifery academization may have been underrepresented through this strategy. Finally, social desirability bias, especially in self-report surveys among professionals identifying with their disciplinary norms, may have contributed to the consistently high relevance ratings [94]. These alternative interpretations, together with the methodological limitations, indicate that the null results may have several distinct explanations and cannot, based on this study, be interpreted as evidence of substantive stakeholder agreement.

### 4.2. Limitations

When interpreting the results of the mixed MANOVA, several methodological limitations should be considered. Despite the skewness and kurtosis values remaining within acceptable thresholds and no detection of outliers, significant deviations from normality were observed (Shapiro–Wilk tests for most variables *p* < 0.001) and the clustering of the data towards the higher end of the scale is consistent with a potential ceiling effect. This restriction of variance, together with a tendency to rate items as highly relevant (response bias [80]), may have limited the statistical sensitivity to detect meaningful differences among groups and may have contributed to the non-significant between-group results [79]. Respondents may have been influenced by social desirability in their responses [80] and therefore may have felt reluctant to rate the competency goals surveyed as irrelevant. No separate statistical analysis of response bias was conducted; this should be considered when interpreting the results.

Although the realized sample size of N = 120 exceeded the a priori target, the distribution across subgroups was uneven (midwives applying professionals (*n* = 48), applying professionals from related professions (*n* = 6), midwife educators (*n* = 41), educators from related professions (*n* = 25). The strongly underpopulated cell of applying professionals from related professions (*n* = 6) reduced statistical power for detecting interaction effects involving this subgroup. The N = 40 estimate should therefore be understood as a general planning threshold under idealized conditions rather than a guarantee of adequate power across all subgroup comparisons. A Type II error (failing to detect effects that are real but small) cannot be ruled out [79]. Future research should aim for larger and more balanced samples across all subgroups to enable adequately powered tests for interaction effects.

A significant violation of the homogeneity of variance-covariance matrices (Box’s M test) was detected. As the Box’s M test is sensitive to deviations from multivariate normality and unequal subgroup sizes, this test statistic should be interpreted with caution [79]. While the a priori defined contrasts were theoretically derived and justified, their interpretation requires caution in the absence of significant multivariate main effects [79].

As the data were collected with a single measurement point, the internal validity of this cross-sectional study is limited [95]. External validity is also constrained due to the active involvement of the researcher in the recruitment process and the combination of non-probability sampling strategies (self-selection, snowball and convenience sampling), with potential selection bias due to voluntary participation reinforced by the online format [80,96].

The sample was not heterogeneous, as more than half of the participants were studying or working in Germany, limiting the representativeness of educational and healthcare system from other included OECD countries [3,43]. As a result of the recruitment strategy (focus on professional networks in Germany), the findings primarily reflect the perspectives of stakeholders embedded in the German midwifery education and healthcare system. The results cannot be generalized beyond the German context without replication in samples with a broader and more balanced study population. Future studies should focus on international recruitment strategies to ensure adequate representation.

Although the questionnaire underwent psychometric validation, additional quality criteria are still pending [31]. The German translation did not follow the recommended procedure for the cultural adaptation of validated instruments, which may have led to cultural bias [81].

The discriminatory capacity of the four control items designed to distinguish Bachelor’s from Master’s competencies is limited. The items yielded mean scores above the scale midpoint, indicating that participants perceived Bachelor’s competencies as relevant as at Master’s level. This suggests limited distinction between Bachelor’s and Master’s level items, which may also reflect a broader overlap in how these qualification levels are conceptualized in practice, limiting the study’s ability to capture qualification-level distinctions.

Another limitation is the low response rate among students in midwifery Master’s programs (*n* = 21). As no publicly available contact information existed for this group, recruitment relied on snowball sampling, with educators forwarding the survey invitation to potential participating students. This approach was not sufficient to achieve a sample size large enough to include students as a separate group in the statistical analyses. Students were combined with practitioners into the “applying professionals” group based on their shared practice-oriented function perspectives. Future research should include a sufficient number of midwifery Master’s students to allow for a separate subgroup analysis.

### 4.3. Implications for Policy, Practice and Research

Policy: The non-significant results do not yet provide an empirical basis for the development of comparable international standards or competency frameworks for midwifery Master’s programs. Rather, they identify open questions for future research. For several years, international organizations have emphasized the need for harmonized global competency frameworks to enhance the quality of midwifery education and the professionalization of midwifery practice [3,97]. However, progress at the Master’s level remains limited [19]. This study can serve as a starting point for decision-makers in education and health policy to further promote the academization of the midwifery profession and strengthen the role of specialized professionals educated at the Master’s level (e.g., AMP) [19,39,98]. Combined with the significant within-subject effect across competency facets and the descriptive patterns, the absence of significant main and interaction effects in the mixed MANOVA supports not fully standardizing Master’s curricula at the international level. Rather, they suggest defining a limited set of academic core competencies, complemented by optional specialized domains (e.g., AMP and midwifery educator). This allows countries and institutions to align their programs with national regulations, workforce needs and healthcare settings, while maintaining international comparability at the level of core competencies [16,31,43].

Nevertheless, the findings should first be re-evaluated in the context of the methodological limitations and complemented by additional stakeholder perspectives [44]. Further empirical evidence is required before the results can be used as a basis for educational and health policy decisions [49,88].

Practice: For professional midwifery practice, the results indicate no statistically detectable group-based differences in midwifery Master’s competencies, while suggesting context-specific relevance of specialized domains (AMP, midwifery educator). The small effect sizes (η^2^_p_ = 0.008–0.012) for the main effects further suggest that differences among profession and function are of limited practical relevance. While the interaction effect profession x function x competency facets (η^2^_p_ = 0.174) exceeded the predefined threshold for large effects (η^2^_p_ > 0.14), this must be interpreted with caution due to the small subgroup sizes. However, the large and significant within-subject effect (η^2^_p_ = 0.492) highlights meaningful differences among the competency facets rather than among stakeholder groups. Simultaneously, the ongoing challenge of theory–practice transfer remains [59], as Master’s programs primarily focus on theoretical and research-oriented competencies [19]. Their practical implementation in clinical settings depends on appropriate structural and organizational conditions [99]. Where midwives with Master’s degrees are granted access to career pathways in leadership, research, and education, the absence of statistically significant group differences on competency goals may not only contribute to improved clinical practice but could also support the long-term professionalization of the midwifery profession [2,98]. The practical translation of these findings is constrained by structural tensions, including the limited time scope of midwifery Master’s programs [10], heterogenous national regulatory frameworks [9,10,11,12,13,14,15] and the persistent gap between academic and clinical environments [44]. These tensions must be addressed in future research, as they extend beyond the scope of the present study.

From a healthcare perspective, qualified midwives with Master’s-level competencies contribute to midwifery leadership [38], research [32], education [25] and advanced clinical roles [39,98] that are essential for strengthening health systems and improving population health outcomes [3].

Research: The results of the statistical analyses provide a starting point for future competency research on an international Master’s competency framework, pending replication with a broader international sample. However, a re-examination with larger and more balanced samples could confirm or expand the results for the identification of potentially subtle group differences, as even small differences in stakeholder perceptions may influence the participatory development of curricula and the implementation of competency goals [46,47]. Future research should also aim for a more international and diverse sample to ensure the generalizability of the results beyond the German context. Furthermore, future studies should focus on students enrolled in midwifery Master’s programs, as they are highly relevant to the research question [100]; however, due to the limitations in the recruitment process (see Section 2), there were not enough students involved in this study. Nevertheless, a separate analysis of students is recommended for future research. In addition, further political and organizational stakeholders should be included to capture diverse perspectives on competency goals [44]. A future qualitative study could provide more detailed feedback on each item/competency goal and thereby strengthen the content validity of the survey instrument [80], allowing for a re-evaluation of if a practical consensus of stakeholders on competency goals exists.

## 5. Conclusions

The results of this cross-sectional study revealed no significant multivariate differences among profession (midwives vs. related professions) and function (midwifery educator vs. applying professionals) or interaction effect, with a significant within-subject effect across competency facets. Stakeholders demonstrated high ratings across all competency facets, particularly general academic competencies. The findings reveal no statistically detectable role- or profession-specific differences in the assessed relevance of Master’s competencies in midwifery. However, the absence of detectable differences cannot be interpreted as evidence of consensus among stakeholder groups, given several possible explanations as well as methodological limitations. These methodological limitations, such as unequal subgroup sample sizes for interaction effects or the underrepresentation of midwifery Master’s students, should be addressed in future research. The results make a contribution to future discussions on competency goals in midwifery Master’s programs, while acknowledging that the predominantly German sample limits the generalizability of these findings. General academic competencies received the highest relevance ratings across stakeholder groups, while specialized competencies for AMP and midwifery educators demonstrated more differentiated rating patterns. Overall, this study provides a preliminary starting point for future research on international competency frameworks on midwifery Master’s programs, pending replication with more representative samples.

## Figures and Tables

**Table 1:** Overview of the factorial structure of the competency domains by Kranz et al. [19,31].

Competency Facet/Factor	Number of Items	Example Item
Competency domain I: General competencies for midwifery Master’s programs		
F1_F1 Ability to promote evidence-based practice	5	I-1-1 Ability to identify gaps between evidence and practice
F1_F2 Ability to initiate and coordinate midwifery research and to evaluate and apply best-practice models	4	I-2-2 Ability to initiate and coordinate research for advancing high-quality health care
F1_F3 Ability to analyze health policy	3	I-3-1 Ability to analyze the process of health policy development
F1_F4 Ability to work in interprofessional collaboration	2	I-4-1 Ability to work as an effective team member in interprofessional collaboration
F1_F5 Ability to identify midwifery theories	4	I-5-1 Ability to identify relevant theories to midwifery practice
F1_F6 Ability to use technologies	2	I-6-1 Ability to improve the quality of health care practice using technologies
F1_F7 Ability to manage finances and resources	3	I-7-1 Ability to evaluate financial aspects of health care practice
F1_F8 Ability to demonstrate the professional understanding of the midwifery profession in different contexts	5	I-8-1 Ability to demonstrate the professional understanding of the midwifery profession in clinical practice
Competency domain II: Specific competencies for Advanced Midwifery Practice		
F2_F1 Ability to respect dignity, trust and discretion	2	II-1-1 Ability to apply ethically sound solutions to complex issues related to the care of women and their babies
F2_F2 Ability to assume professional responsibility and accountability in a leadership role	2	II-2-1 Ability to use advanced knowledge, skills and abilities for engaging in a leadership role in complex clinical decision-making
F2_F3 Ability to protect and promote quality of practice	3	II-3-1 Ability to promote and protect high-quality maternity care and services
F2_F4 Ability for collaborative risk assessment	2	II-4-1 Ability to manage collaborative risk assessment with others
Competency domain III: Specific competencies for midwifery educator		
F3_F1 Ability to incorporate and promote ethical and legal principles into teaching	3	III-1-1 Ability to integrate ethical aspects of midwifery care into teaching/learning activity
F3_F2 Ability to maintain and update midwifery competencies in theory and practice	2	III-2-1 Ability to maintain knowledge and skills of theory and practice in midwifery up to date
F3_F3 Ability to create a conducive environment for theoretical learning	3	III-3-1 Ability to incorporate educational strategies for promoting active learning
F3_F4 Ability to create an effective learning environment for clinical teaching	2	III-4-1 Ability to create a safe and effective learning environment in the clinical setting of midwifery care
F3_F5 Ability for quality assurance in educational programs	4	III-5-1 Ability to regular monitor, assess and evaluate a midwifery education program
F3_F6 Ability to advocate and act as a leader	4	III-6-1 Ability to use a variety of communication methods in different settings
F3_F7 Ability to incorporate research culture into teaching	3	III-7-1 Ability to use research findings to inform teaching and practice

**Table 2:** Mixed MANOVA: main effects and interaction effects across all competency facets (N = 120).

Effect	Hypothesis	Pillai’s Trace	F	Df ^1^	*p* ^2^	η^2^_p_ ^3^
Competency facets	-	0.492	5.335	18, 99	<0.001	0.492
Profession (main effect)	1	0.012	1.401	1, 116	0.239	0.012
Function (main effect)	2	0.008	0.975	1, 116	0.325	0.008
Competency facets x profession x function (interaction effect)	4	0.174	1.157	18, 99	0.311	0.174

^1^ Df: Degrees of freedom reported as hypothesis df, error df. ^2^ Significance level was set at *p* < 0.05. ^3^ η^2^_p_ = partial eta squared. Note: The numerical equivalence of Pillai’s Trace and η^2^_p_ across all effects reflects their conceptual relatedness as measures of explained multivariate variance in this design [82].

**Table 3:** A priori contrasts H1: Midwives (*n* = 89) vs. related professions (*n* = 31) (EMMEANS, Bonferroni-corrected).

Hypothesis	Facet	$\mathrm{Midwives}\;x^{-}$ (SE ^1^)	$\mathrm{Related}\;\mathrm{Professions}\;x^{-}$ (SE ^1^)	MD ^2^ (95% CI ^3^)	*p* ^4^ (Bonf.)
H1a	F1_F1 Ability to promote evidence-based practice	4.485 (0.061)	4.310 (0.103)	0.176 [−0.062, 0.414]	0.146
H1a	F1_F2 Ability to initiate and coordinate midwifery research and to evaluate and apply best-practice models	4.281 (0.075)	4.065 (0.127)	0.216 [−0.075, 0.507]	0.144
H1a	F1_F3 Ability to analyze health policy	4.037 (0.094)	3.849 (0.158)	0.188 [−0.176, 0.552]	0.309
H1a	F1_F5 Ability to identify midwifery theories	4.090 (0.085)	3.863 (0.145)	0.227 [−0.105, 0.559]	0.179
H1a	F1_F8 Ability to demonstrate the professional understanding of the midwifery profession in different contexts	3.899 (0.089)	3.723 (0.151)	0.176 [−0.171, 0.524]	0.317
H1a	F2_F3 Ability to protect and promote quality of practice	3.753 (0.132)	3.699 (0.223)	0.054 [−0.460, 0.567]	0.836
H1b	F1_F4 Ability to work in interprofessional collaboration	3.961 (0.110)	4.097 (0.186)	−0.136 [−0.564, 0.292]	0.530
H1b	F1_F6 Ability to use technologies	3.522 (0.111)	3.597 (0.188)	−0.074 [−0.506, 0.357]	0.734
H1b	F1_F7 Ability to manage finances and resources	3.798 (0.093)	3.925 (0.158)	−0.127 [−0.490, 0.237]	0.490

^1^ SE = standard error; ^2^ MD = mean difference; ^3^ CI = confidence interval; ^4^ Significance level was set at *p* < 0.05.

**Table 4:** A priori contrasts H2: educators (*n* = 66) vs. applying professionals (*n* = 54) (EMMEANS, Bonferroni-corrected).

Hypothesis	Facet	$\mathrm{Educators}\;x^{-}$ (SE ^1^)	$\mathrm{Applying}\;\mathrm{Professionals}\;x^{-}$ (SE ^1^)	MD ^2^ (95% CI ^3^)	*p* ^4^ (Bonf.)
H2a	F3_F3 Ability to create a conducive environment for theoretical learning	3.808 (0.136)	3.568 (0.150)	0.240 [−0.161, 0.642]	0.239
H2a	F3_F4 Ability to create an effective learning environment for clinical teaching	3.773 (0.133)	3.620 (0.147)	0.152 [−0.240, 0.545]	0.443
H2a	F3_F5 Ability for quality assurance in educational programs	3.777 (0.119)	3.787 (0.132)	−0.011 [−0.363, 0.342]	0.953
H2a	F3_F7 Ability to incorporate research culture into teaching	4.258 (0.114)	4.173 (0.126)	0.085 [−0.252, 0.422]	0.619
H2b	F2_F2 Ability to assume professional responsibility and accountability in a leadership role	4.258 (0.121)	4.065 (0.133)	0.193 [−0.163, 0.549]	0.286
H2b	F2_F4 Ability for collaborative risk assessment	3.712 (0.137)	3.694 (0.151)	0.018 [−0.387, 0.422]	0.931
H2b	F3_F2 Ability to maintain and update midwifery competencies in theory and practice	4.121 (0.146)	3.981 (0.162)	0.140 [−0.292, 0.571]	0.523
H2b	F3_F6 Ability to advocate and act as a leader	3.962 (0.099)	3.778 (0.109)	0.184 [−0.108, 0.476]	0.214

^1^ SE = standard error; ^2^ MD = mean difference; ^3^ CI = confidence interval; ^4^ significance level was set at *p* < 0.05.

**Table 5:** Tests of between-subjects effects H3: profession and function.

Effect	Facet	$\mathrm{Group}\;1\;x^{-}$ (SE)	$\mathrm{Group}\;2\;x^{-}$ (SE)	F_(df=1,116)_	*p* (Bonf.)	η^2^_p_	MD (95% CI)
Profession	F2_F1 Ability to respect dignity, trust and discretion	Midwives: 3.957 (0.121)	Related professions: 3.963 (0.260)	0.000	0.982	0.000	−0.006 [−0.574, 0.561]
Function		Educators: 3.978 (0.145)	Applying prof.: 3.943 (0.247)	0.015	0.903	0.000	0.035 [−0.533, 0.602]
Profession	F3_F1 Ability to incorporate and promote ethical and legal principles into teaching	Midwives: 4.084 (0.100)	Related professions: 3.798 (0.214)	1.467	0.228	0.012	0.286 [−0.181, 0.753]
Function		Educators: 3.927 (0.119)	Applying prof.: 3.955 (0.204)	0.014	0.905	0.000	−0.028 [−0.496, 0.439]

## Data Availability

The data presented in this study are available on request from the corresponding author. They were collected with informed consent under GDPR. Due to the inclusion of personally identifiable information, the dataset has been pseudonymized but not fully anonymized. In accordance with data protection regulations, the raw data cannot be shared publicly. However, aggregated data or specific analyses may be made available upon reasonable request.

## Supplementary Materials

The following supporting information can be downloaded at: https://www.mdpi.com/article/10.3390/healthcare14101377/s1.

## Abbreviations

The following abbreviations are used in this manuscript:

AMP	Advanced Midwifery Practice
ANOVA	Analysis of Variance
Bonf.	Bonferroni-adjusted
CBE	Competency-Based Education
CFA	Confirmatory Factor Analysis
CI	Confidence Interval
Df	Degrees of Freedom
EMMEANS	Estimated Marginal Means
EU	European Union
F	F-statistic
G*Power	Statistical Power Analysis Software
GDPR	General Data Protection Regulation
ICM	International Confederation of Midwives
MANOVA	Multivariate Analysis of Variance
MD	Mean Difference
OECD	Organization for Economic Co-operation and Development
*p*	Probability value
r	Pearson’s correlation coefficient
SE	Standard Error
SPSS	Statistical Package for the Social Sciences
η^2^_p_	Partial Eta Squared

## Appendix A

**Table A1:** Construct-level intercorrelations (*n* = 120).

		Construct I (General Competencies)	Construct II (AMP Competencies)	Construct III (Midwifery Educator Competencies)
Construct I (general competencies)	Pearson correlation	1	0.463 **	0.564 **
	Sig. (two-tailed)		<0.001	<0.001
Construct II (AMP competencies)	Pearson correlation	0.463 **	1	0.671 **
	Sig. (two-tailed)	<0.001		<0.001
Construct III (midwifery educator competencies)	Pearson correlation	0.564 **	0.671 **	1
	Sig. (two-tailed)	<0.001	<0.001	

** = Significance level: *p* < 0.001; r = 0.10 = small, 0.30 = medium, 0.50 = large; Note: Correlations are based on mean scale scores calculated for each competency domain.

**Table A2:** Socio-demographic characteristics of study participants (*n* = 120) (previously published by Kranz et al. [31]).

Characteristics	*n* (%)
Gender	
Female	114 (95.0%)
Male	5 (4.2%)
Other	1 (0.8%)
Age (years)	
18–30	28 (23.2%)
31–40	26 (21.7%)
41–50	27 (22.5%)
51–60	31(25.8%)
61–70	8 (6.6%)
Country of work or study	
Australia	1 (0.8%)
Austria	3 (2.5%)
Belgium	6 (5.0%)
Canada	2 (1.7%)
Germany	77 (64.2%)
England	4 (3.3%)
Estonia	4 (3.3%)
Ireland	1 (0.8%)
Italy	5 (4.2%)
Japan	1 (0.8%)
Kingdom of Saudi Arabia	1 (0.8%)
Luxembourg	2 (1.7%)
Netherlands	1 (0.8%)
Norway	1 (0.8%)
Switzerland	6 (5.0%)
Scotland	1 (0.8%)
South Africa	1 (0.8%)
Sweden	2 (1.7%)
Turkey	1 (0.8%)
Professional degree (several options possible)	
Completed vocational training in midwifery	52 (43.3%)
Completed vocational training in nursing	6 (5.0%)
Completed Bachelor’s degree (or equivalent) in midwifery science	51 (42.5%)
Completed Master’s degree (or equivalent) in midwifery science	25 (20.8%)
Completed Bachelor’s degree (or equivalent) in nursing science	12 (10.0%)
Completed Master’s degree (or equivalent) in nursing science	7 (5.8%)
Completed Bachelor’s degree in related profession	6 (5.0%)
Completed Master’s degree in related profession	21 (17.5%)
Doctorate	20 (16.7%)
Habilitation	4 (3.3%)
Other professional degree	11 (9.2%)
Higher education of parents	
Yes	60 (50.0%)
No	60 (50.0%)
Study participants characteristics	
Student in Master’s program in midwifery science	21 (17.5%)
Lecturer in Master’s program midwifery science	33 (27.5%)
Working in a professional environment outside of higher education related to midwifery	33 (27.5%)
Lecturer in Bachelor’s program midwifery science	33 (27.5%)

**Table A3:** Descriptive statistics on item-level, grouped by factors/competency facets (Table published in preliminary work, modified according to Kranz et al. [31]) (N = 120).

Items *	$x^{-}$ Item ^1^	σ ^2^
Construct I: General competencies for midwifery Master’s programs		
Construct I, factor/competency facet 1: Ability to promote evidence-based practice		
I-1-1 Identifying evidence-practice gaps	4.33	0.909
I-1-2 Bridging evidence-practice gaps	4.46	0.732
I-1-3 Evidence-based decision-making	4.56	0.646
I-1-4 Promotions of evidence-based midwifery practice	4.43	0.786
I-1-5 Assessment and utilization of research findings	4.42	0.796
Construct I, factor/competency facet 2: Ability to initiate and coordinate midwifery research and to evaluate and apply best-practice models		
I-2-1 Evaluation and use of research for practice improvement **	4.43	0.730
I-2-4 Integrating clinical expertise in best-practice models **	4.11	0.807
I-2-2 Initiation and coordination of research to advance care	4.23	0.923
I-2-3 Development of research-based care models	4.31	0.924
I-2-5 Use data to analyze outcomes	4.16	0.860
I-2-6 Connecting data to best-practice models	4.20	0.836
Construct I, factor/competency facet 3: Ability to analyze health policy		
I-3-1 Analyzing health policy development	3.85	0.984
I-3-2 Analyzing influential factors on health policy	4.01	0.930
I-3-3 Analyzing policy effects on clinical practice	4.11	0.915
Construct I, factor/competency facet 4: Ability to work in interprofessional collaboration		
I-4-1 Effective interprofessional teamwork	3.88	1.168
I-4-2 Improving interprofessional collaboration for positive change	4.11	1.044
Construct I, factor/competency facet 5: Ability to identify midwifery theories		
I-5-1 Identifying theories relevant to midwifery practice	3.99	0.974
I-5-2 Identifying theories relevant to midwifery science	4.07	0.945
I-5-3 Identifying theories relevant to health equity and social justice	4.00	0.987
I-5-4 Identifying theories relevant to ethics in midwifery care	4.06	0.973
Construct I, factor/competency facet 6: Ability to use technologies:		
I-6-1 Improving health care quality using technologies	3.55	1.076
I-6-2 Improving health care safety using technologies	3.53	1.061
Construct I, factor/competency facet 7: Ability to manage finances and resources		
I-7-1 Evaluation of health care finances	3.72	1.069
I-7-2 Use of health care resources	3.93	0.945
I-7-3 Management of health care resources	3.84	0.961
Construct I, factor/competency facet 8: Ability to demonstrate the professional understanding of the midwifery profession in different contexts		
I-8-1 Demonstrating professional understanding in clinical practice	3.87	1.216
I-8-2 Demonstrating professional understanding in administration	3.48	1.115
I-8-3 Demonstrating professional understanding in policy implementation	3.95	0.969
I-8-4 Demonstrate health disparities in midwifery practice	3.91	1.077
I-8-5 Exploration of areas of interest in midwifery	4.06	0.981
Construct II: Specific competencies for Advanced Midwifery Practice		
Construct II, factor/competency facet 1: Ability to respect the dignity, trust and discretion		
II-1-1 Applying ethical solutions to complex issues in maternal and newborn care	3.96	1.177
II-1-2 Advocacy and negotiation for women, their babies and family’s rights	3.87	1.341
Construct II, factor/competency facet 2: Ability to assume professional responsibility and accountability in a leadership role		
II-2-1 Leadership in complex clinical decision-making	4.13	1.020
II-2-2 Leadership in problem-solving	4.21	0.978
Construct II, factor/competency facet 3: Ability to protect and promote quality of practice		
II-3-1 Promoting high-quality maternity care	3.83	1.311
II-3-2 Promoting compassionate maternity care	3.45	1.466
II-3-3 Promoting evidence-based safe maternity care	3.93	1.207
Construct II, factor/competency facet 4: Ability for collaborative risk assessment		
II-4-1 Collaborative risk management	3.79	1.137
II-4-2 Collaborative safety promotion	3.62	1.210
Construct III: Specific competencies for midwifery educator		
Construct III, factor/competency facet 1: Ability to incorporate and promote ethical and legal principles into teaching		
III-1-1 Integrating ethical aspects into teaching	3.94	1.031
III-1-2 Integrating legal aspects into teaching	3.97	1.045
III-1-3 Role modeling ethical and legal aspects in teaching	4.07	0.950
Construct III, factor/competency facet 2 Ability to maintain and update midwifery competencies in theory and practice		
III-2-1 Maintaining midwifery knowledge and skills up to date	3.99	1.267
III-2-2 Evidence-based midwifery education	4.13	1.206
Construct III, factor/competency facet 3 Ability to create a conducive environment for theoretical learning		
III-3-1 Promoting active learning	3.68	1.124
III-3-2 Using effective teaching and learning materials	3.73	1.130
III-3-3 Recognizing and supporting learning styles and needs	3.70	1.149
Construct III, factor/competency facet 4 Ability to create an effective learning environment for clinical teaching		
III-4-1 Creating safe clinical learning environments	3.77	1.106
III-4-2 Promoting experiential learning	3.64	1.172
Construct III, factor/competency facet 5: Ability for quality assurance in educational programs		
III-5-1 Monitoring and evaluating midwifery programs	3.69	1.114
III-5-2 Assessment of student competencies	3.69	1.106
III-5-3 Participation in the midwifery curriculum	3.87	1.100
III-5-4 Revision and implementation of midwifery programs	3.88	1.034
Construct III, factor/competency facet 6: Ability to advocate and act as a leader		
III-6-1 Using varied communication methods	3.53	1.202
III-6-2 Advocating change in midwifery practice and education	4.06	0.964
III-6-3 Advocacy in midwifery practice and education	3.87	0.970
III-6-4 Leadership in practice and education	4.06	0.990
Construct III, factor/competency facet 7: Ability to incorporate research culture into teaching		
III-7-1 Using research to inform teaching and practice	4.18	1.066
III-7-2 Supporting evidence-based culture	4.25	0.946
III-7-3 Promoting critical inquiry	4.23	1.098
Control items with general midwifery Bachelor’s abilities (no construct assigned)		
C-1 Upholding human rights in midwifery care	3.56	1.471
C-2 Adhering to laws and professional standards in midwifery	3.40	1.475
C-3 Facilitating normal birth across settings	3.57	1.505
C-4 Recognizing and referring beyond midwifery scope	3.41	1.487

^1^$\;x^{-}$ item = arithmetic mean for all items: 1.00 “ Not important in the Master’s program” to 5.00 “Very important in the Master’s program”; ^2^ σ = standard deviation for all items; * Short version of the items, for detailed description of the items please refer to the Supplementary Materials S3 and S4; ** Items excluded in the CFA (for details Kranz et al. [31]).

**Table A4:** Descriptive statistics on factor-level (means and standard deviations) of the 19 competency facets by profession and function.

Factor/Competency Facet	$\mathrm{All}\;\mathrm{Participants}\;(n\;=\;120)\;x^{-}$ (σ)	Midwives (*n* = 89) $\;x^{-}$ (σ)	Related Professions (*n* = 31) $\;x^{-}$ (σ)	Midwifery Educator (*n* = 41) $\;x^{-}$ (σ)	Related Profession Educators (*n* = 25) $\;x^{-}$ (σ)	Midwives Applying Professionals (*n* = 48) $\;x^{-}$ (σ)	Related Professions Applying Professionals (*n* = 6) $\;x^{-}$ (σ)
Construct 1: General competencies for midwifery Master’s programs							
F1_F1 Ability to promote evidence-based practice	4.44 (0.579)	4.486 (0.530)	4.310 (0.694)	4.571 (0.468)	4.344 (0.706)	4.413 (0.572)	4.167 (0.686)
F1_F2 Ability to initiate and coordinate midwifery research and to evaluate and apply best-practice models	4.225 (0.708)	4.281 (0.630)	4.065 (0.887)	4.378 (0.602)	4.120 (0.842)	4.198 (0.648)	3.833 (1.114)
F1_F3 Ability to analyze health policy	3.989 (0.882)	4.038 (0.826)	3.850 (1.029)	4.073 (0.762)	3.960 (1.042)	4.007 (0.885)	3.389 (0.905)
F1_F4 Ability to work in interprofessional collaboration	3.996 (1.034)	3.961 (1.015)	4.097 (1.099)	3.927 (1.046)	4.220 (1.021)	3.990 (0.997)	3.583 (1.357)
F1_F5 Ability to identify midwifery theories	4.031 (0.807)	4.090 (0.761)	3.863 (0.922)	4.177 (0.765)	3.930 (0.885)	4.016 (0.757)	3.583 (1.103)
F1_F6 Ability to use technologies	3.542 (1.040)	3.523 (1.005)	3.597 (1.150)	3.585 (1.042)	3.620 (1.073)	3.469 (0.981)	3.500 (1.549)
F1_F7 Ability to manage finances and resources	3.831 (0.878)	3.798 (0.881)	3.925 (0.876)	3.683 (0.957)	3.973 (0.907)	3.896 (0.808)	3.722 (0.772)
F1_F8 Ability to demonstrate the professional understanding of the midwifery profession in different contexts	3.853 (0.841)	3.899 (0.831)	3.723 (0.870)	3.888 (0.880)	3.704 (0.796)	3.908 (0.796)	3.800 (1.220)
Construct II: Specific competencies for Advanced Midwifery Practice							
F2_F1 Ability to respect dignity, trust and discretion	3.912 (1.150)	3.938 (1.123)	3.839 (1.241)	4.195 (1.123)	3.760 (1.243)	3.719 (1.086)	4.167 (1.291)
F2_F2 Ability to assume professional responsibility and accountability in a leadership role	4.171 (0.980)	4.185 (1.010)	4.129 (0.903)	4.293 (0.790)	4.200 (0.890)	4.094 (1.165)	3.833 (0.983)
F2_F3 Ability to protect and promote quality of practice	3.740 (1.238)	3.753 (1.243)	3.699 (1.245)	3.805 (1.287)	3.627 (1.233)	3.708 (1.216)	4.000 (1.366)
F2_F4 Ability for collaborative risk assessment	3.704 (1.109)	3.730 (1.087)	3.629 (1.183)	3.731 (1.168)	3.680 (1.215)	3.729 (1.026)	3.417 (1.114)
Construct III: Specific competencies for midwifery educator							
F3_F1 Ability to incorporate and promote ethical and legal principles into teaching	3.992 (0.942)	4.079 (0.935)	3.742 (0.934)	4.146 (1.014)	3.710 (0.935)	4.021 (0.868)	3.889 (1.004)
F3_F2 Ability to maintain and update midwifery competencies in theory and practice	4.058 (1.185)	3.989 (1.257)	4.258 (0.939)	4.061 (1.347)	4.220 (0.936)	3.927 (1.185)	4.417 (1.021)
F3_F3 Ability to create a conducive environment for theoretical learning	3.700 (1.107)	3.787 (1.101)	3.452 (1.104)	3.902 (1.114)	3.653 (0.945)	3.688 (1.092)	2.611 (1.405)
F3_F4 Ability to create an effective learning environment for clinical teaching	3.704 (1.078)	3.753 (1.077)	3.565 (1.086)	3.842 (1.092)	3.660 (1.038)	3.677 (1.069)	3.167 (1.291)
F3_F5 Ability for quality assurance in educational programs	3.873 (0.806)	3.874 (0.928)	3.516 (1.035)	3.896 (0.986)	3.580 (1.007)	3.854 (0.886)	3.250 (1.204)
F3_F6 Ability to advocate and act as a leader	3.879 (0.806)	3.921 (0.820)	3.758 (0.762)	4.085 (0.788)	3.760 (0.772)	3.781 (0.829)	3.750 (0.790)
F3_F7 Ability to incorporate research culture into teaching	4.219 (0.924)	4.281 (0.861)	4.043 (1.081)	4.439 (0.751)	3.960 (1.103)	4.146 (0.933)	4.389 (0.998)

$x^{-}$ = arithmetic mean for all items: 1.00 “ Not important in the Master’s program” to 5.00 “Very important in the Master’s program”; σ = standard deviation.

**Table A5:** Univariate test for normality (Shapiro–Wilk), skewness/kurtosis, Levene-test and univariate outliers (Q-Q) per facet and group.

Competency Facet	Group	Shapiro–Wilk (*p*=)	Skewness (SE)	Kurtosis (SE)	Levene-Test (*p*=)	Univariate Outliers (Q-Q)
Construct 1: General competencies for midwifery Master’s programs						
F1_F1	Midwives (*n* = 89)	<0.001	−1.176 (0.255)	0.856 (0.506)	0.572	None
	Related profession (*n* = 31)	<0.001	−1.681 (0.421)	4.594 (0.821)		None
	Midwifery educator (*n* = 41)	<0.001	−1.106 (0.369)	0.439 (0.724)		None
	Related profession educator (*n* = 25)	<0.001	−1.941 (0.464)	6.069 (0.902)		None
	Midwives applying professionals (*n* = 48)	<0.001	−1.140 (0.343)	0.717 (0.674)		None
	Related professions applying professionals (*n* = 6)	0.683	−0.859 (0.845)	1.254 (1.741)		None
F1_F2	Midwives (*n* = 89)	<0.001	−0.830 (0.255)	0.243 (0.506)	0.063	None
	Related profession (*n* = 31)	<0.001	−1.078 (0.421)	0.122 (0.821)		None
	Midwifery educator (*n* = 41)	<0.001	−1.385 (0.369)	2.608 (0.724)		None
	Related profession educator (*n* = 25)	0.002	−1.329 (0.464)	1.167 (0.902)		None
	Midwives applying professionals (*n* = 48)	0.005	−0.464 (0.343)	−0.746 (0.674)		None
	Related professions applying professionals (*n* = 6)	0.176	−0.452 (0.845)	−2.129 (1.741)		None
F1_F3	Midwives (*n* = 89)	<0.001	−0.681 (0.255)	−0.017 (0.506)	0.041 *	None
	Related profession (*n* = 31)	0.004	−0.366 (0.421)	−1.155 (0.821)		None
	Midwifery educator (*n* = 41)	<0.001	−0.680 (0.369)	0.171 (0.724)		None
	Related profession educator (*n* = 25)	0.002	−0.475 (0.464)	−1.184 (0.902)		None
	Midwives applying professionals (*n* = 48)	0.001	−0.665 (0.343)	−0.140 (0.674)		None
	Related professions applying professionals (*n* = 6)	0.459	−0.712 (0.845)	−0.955 (1.741)		None
F1_F4	Midwives (*n* = 89)	<0.001	−0.742 (0.255)	−0.290 (0.506)	0.611	None
	Related profession (*n* = 31)	<0.001	−0.928 (0.421)	−0.594 (0.821)		None
	Midwifery educator (*n* = 41)	<0.001	−0.536 (0.369)	−0.826 (0.724)		None
	Related profession educator (*n* = 25)	<0.001	−1.133 (0.464)	−0.049 (0.902)		None
	Midwives applying professionals (*n* = 48)	<0.001	−0.961 (0.343)	0.394 (0.674)		None
	Related professions applying professionals (*n* = 6)	0.191	−0.262 (0.845)	−1.987 (1.741)		None
F1_F5	Midwives (*n* = 89)	<0.001	−0.639 (0.255)	−0.185 (0.506)	0.568	None
	Related profession (*n* = 31)	0.027	−0.556 (0.421)	−0.434 (0.821)		None
	Midwifery educator (*n* = 41)	0.001	−0.837 (0.369)	−0.067 (0.724)		None
	Related profession educator (*n* = 25)	0.052	−0.642 (0.464)	−0.114 (0.902)		None
	Midwives applying professionals (*n* = 48)	0.012	−0.513 (0.343)	−0.037 (0.674)		None
	Related professions applying professionals (*n* = 6)	0.954	−0.208 (0.845)	−0.811 (1.741)		None
F1_F6	Midwives (*n* = 89)	<0.001	−0.166 (0.255)	−0.810 (0.506)	0.225	None
	Related profession (*n* = 31)	0.018	−0.569 (0.421)	−0.590 (0.821)		None
	Midwifery educator (*n* = 41)	0.004	−0.500 (0.369)	−0.417 (0.724)		None
	Related profession educator (*n* = 25)	0.053	−0.696 (0.464)	0.014 (0.902)		None
	Midwives applying professionals (*n* = 48)	<0.001	0.144 (0.343)	−1.031 (0.674)		None
	Related professions applying professionals (*n* = 6)	0.213	−0.303 (0.845)	−0.303 (1.741)		None
F1_F7	Midwives (*n* = 89)	<0.001	−0.302 (0.255)	−0.794 (0.506)	0.517	None
	Related profession (*n* = 31)	0.022	−0.914 (0.421)	1.020 (0.821)		None
	Midwifery educator (*n* = 41)	0.030	−0.224 (0.369)	−0.900 (0.724)		None
	Related profession educator (*n* = 25)	0.013	−1.215 (0.464)	1.648 (0.902)		None
	Midwives applying professionals (*n* = 48)	0.015	−0.281 (0.343)	−0.851 (0.674)		None
	Related professions applying professionals (*n* = 6)	0.094	1.148 (0.845)	−0.057 (1.741)		None
F1_F8	Midwives (*n* = 89)	<0.001	−0.606 (0.255)	−0.243 (0.506)	0.170	None
	Related profession (*n* = 31)	0.062	0.089 (0.421)	−1.238 (0.821)		None
	Midwifery educator (*n* = 41)	0.021	−0.662 (0.369)	−0.281 (0.724)		None
	Related profession educator (*n* = 25)	0.226	0.188 (0.464)	−1.101 (0.902)		None
	Midwives applying professionals (*n* = 48)	0.034	−0.553 (0.343)	−0.145 (0.674)		None
	Related professions applying professionals (*n* = 6)	0.219	−0.222 (0.845)	−2.380 (1.741)		None
Construct II: Specific competencies for Advanced Midwifery Practice						
F2_F1	Midwives (*n* = 89)	<0.001	−0.810 (0.255)	−0.381 (0.506)	0.688	None
	Related profession (*n* = 31)	<0.001	−0.625 (0.421)	−0.875 (0.821)		None
	Midwifery educator (*n* = 41)	<0.001	−1.240 (0.369)	0.453 (0.724)		None
	Related profession educator (*n* = 25)	0.004	−0.619 (0.464)	−0.737 (0.902)		None
	Midwives applying professionals (*n* = 48)	0.002	−0.594 (0.343)	−0.494 (0.674)		None
	Related professions applying professionals (*n* = 6)	0.001	−0.968 (0.845)	−1.875 (1.741)		None
F2_F2	Midwives (*n* = 89)	<0.001	−1.371 (0.255)	1.639 (0.506)	0.342	None
	Related profession (*n* = 31)	<0.001	−0.558 (0.421)	−0.895 (0.821)		None
	Midwifery educator (*n* = 41)	<0.001	−0.704 (0.369)	−0.804 (0.724)		None
	Related profession educator (*n* = 25)	<0.001	−0.810 (0.464)	−0.278 (0.902)		None
	Midwives applying professionals (*n* = 48)	<0.001	−1.383 (0.343)	1.215 (0.674)		None
	Related professions applying professionals (*n* = 6)	0.035	0.456 (0.845)	−2.390 (1.741)		None
F2_F3	Midwives (*n* = 89)	<0.001	−0.687 (0.255)	−0.556 (0.506)	0.871	None
	Related profession (*n* = 31)	0.003	−0.762 (0.421)	−0.458 (0.821)		None
	Midwifery educator (*n* = 41)	<0.001	−0.716 (0.369)	−0.633 (0.724)		None
	Related profession educator (*n* = 25)	0.012	−0.744 (0.464)	−0.306 (0.902)		None
	Midwives applying professionals (*n* = 48)	<0.001	−0.698 (0.343)	−0.390 (0.674)		None
	Related professions applying professionals (*n* = 6)	0.042	−1.307 (0.845)	0.387 (1.741)		None
F2_F4	Midwives (*n* = 89)	<0.001	−0.376 (0.255)	−0.977 (0.506)	0.467	None
	Related profession (*n* = 31)	0.006	−0.523 (0.421)	−0.747 (0.821)		None
	Midwifery educator (*n* = 41)	<0.001	−0.298 (0.369)	−1.439 (0.724)		None
	Related profession educator (*n* = 25)	0.009	−0.667 (0.464)	−0.558 (0.902)		None
	Midwives applying professionals (*n* = 48)	0.003	−0.485 (0.343)	−0.384 (0.674)		None
	Related professions applying professionals (*n* = 6)	0.801	0.148 (0.845)	−1.128 (1.741)		None
Construct III: Specific competencies for midwifery educator						
F3_F1	Midwives (*n* = 89)	<0.001	−1.184 (0.255)	1.195 (0.506)	0.714	None
	Related profession (*n* = 31)	0.007	−0.510 (0.421)	−0.624 (0.821)		None
	Midwifery educator (*n* = 41)	<0.001	−1.445 (0.369)	1.660 (0.724)		None
	Related profession educator (*n* = 25)	0.052	−0.355 (0.464)	−0.781 (0.902)		None
	Midwives applying professionals (*n* = 48)	<0.001	−0.954 (0.343)	1.002 (0.674)		None
	Related professions applying professionals (*n* = 6)	0.075	−1.565 (0.845)	3.609 (1.741)		None
F3_F2	Midwives (*n* = 89)	<0.001	−1.169 (0.255)	0.290 (0.506)	0.246	None
	Related profession (*n* = 31)	<0.001	−1.174 (0.421)	0.441 (0.821)		None
	Midwifery educator (*n* = 41)	<0.001	−1.305 (0.369)	0.416 (0.724)		None
	Related profession educator (*n* = 25)	<0.001	−1.160 (0.464)	0.600 (0.902)		None
	Midwives applying professionals (*n* = 48)	<0.001	−1.090 (0.343)	0.390 (0.674)		None
	Related professions applying professionals (*n* = 6)	0.004	−1.783 (0.845)	2.774 (1.741)		None
F3_F3	Midwives (*n* = 89)	<0.001	−0.607 (0.255)	−0.602 (0.506)	0.541	None
	Related profession (*n* = 31)	0.024	−0.411 (0.421)	−0.614 (0.821)		None
	Midwifery educator (*n* = 41)	<0.001	−0.738 (0.369)	−0.357 (0.724)		None
	Related profession educator (*n* = 25)	0.009	−0.469 (0.464)	−0.456 (0.902)		None
	Midwives applying professionals (*n* = 48)	0.002	−0.538 (0.343)	−0.687 (0.674)		None
	Related professions applying professionals (*n* = 6)	0.602	0.919 (0.845)	0.986 (1.741)		None
F3_F4	Midwives (*n* = 89)	<0.001	−0.654 (0.255)	−0.365 (0.506)	0.835	None
	Related profession (*n* = 31)	0.028	−0.219 (0.421)	−0.966 (0.821)		None
	Midwifery educator (*n* = 41)	<0.001	−0.555 (0.369)	−0.876 (0.724)		None
	Related profession educator (*n* = 25)	0.021	−0.223 (0.464)	−0.987 (0.902)		None
	Midwives applying professionals (*n* = 48)	<0.001	−0.784 (0.343)	0.122 (0.674)		None
	Related professions applying professionals (*n* = 6)	0.945	0.077 (0.845)	−0.867 (1.741)		None
F3_F5	Midwives (*n* = 89)	<0.001	−0.628 (0.255)	−0.498 (0.506)	0.310	None
	Related profession (*n* = 31)	0.143	−0.354 (0.421)	−0.704 (0.821)		None
	Midwifery educator (*n* = 41)	<0.001	−0.510 (0.369)	−1.097 (0.724)		None
	Related profession educator (*n* = 25)	0.340	−0.516 (0.464)	−0.275 (0.902)		None
	Midwives applying professionals (*n* = 48)	0.007	−0.801 (0.343)	0.289 (0.674)		None
	Related professions applying professionals (*n* = 6)	0.485	0.322 (0.845)	−1.355 (1.741)		None
F3_F6	Midwives (*n* = 89)	<0.001	−0.611 (0.255)	0.257 (0.506)	0.928	None
	Related profession (*n* = 31)	0.201	0.005 (0.421)	−0.115 (0.821)		None
	Midwifery educator (*n* = 41)	0.001	−0.472 (0.369)	−1.004 (0.724)		None
	Related profession educator (*n* = 25)	0.273	−0.087 (0.464)	0.062 (0.902)		None
	Midwives applying professionals (*n* = 48)	0.025	−0.735 (0.343)	1.027 (0.674)		None
	Related professions applying professionals (*n* = 6)	0.949	0.569 (0.845)	0.148 (1.741)		None
F3_F7	Midwives (*n* = 89)	<0.001	−1.511 (0.255)	2.340 (0.506)	0.203	None
	Related profession (*n* = 31)	<0.001	−1.026 (0.421)	0.475 (0.821)		None
	Midwifery educator (*n* = 41)	<0.001	−1.356 (0.369)	0.898 (0.724)		None
	Related profession educator (*n* = 25)	0.004	−1.011 (0.464)	0.541 (0.902)		None
	Midwives applying professionals (*n* = 48)	<0.001	−1.519 (0.343)	2.466 (0.674)		None
	Related professions applying professionals (*n* = 6)	0.007	−1.379 (0.845)	0.539 (1.741)		None

SE = standard error; outliers defined as cases > 3 box lengths from median; * significance level *p* < 0.05.

**Table A6:** Intercorrelations among the 19 competency facets (Pearson’s r, N = 120).

		F1_F1	F1_F2	F1_F3	F1_F4	F1_F5	F1_F6	F1_F7	F1_F8	F2_F1	F2_F2	F2_F3	F2_F4	F3_F1	F3_F2	F3_F3	F3_F4	F3_F5	F3_F6	F3_F7
F1_F1	Pearson’s r	1	0.453 **	0.324 **	0.243 **	0.451 **	0.128	0.264 **	0.342 **	0.200 *	0.293 **	0.145	0.150	0.287 **	0.216 *	0.276 **	0.193 *	0.199 *	0.302 **	0.349 **
	Sig. (two-tailed)		<0.001	<0.001	0.007	<0.001	0.162	0.004	<0.001	0.029	0.001	0.114	0.103	0.001	0.018	0.002	0.035	0.030	<0.001	<0.001
F1_F2	Pearson’s r	0.453 **	1	0.654 **	0.303 **	0.534 **	0.448 **	0.507 **	0.340 **	0.146	0.383 **	0.047	0.120	0.292 **	0.047	0.392 **	0.221 *	0.498 **	0.460 **	0.284 **
	Sig. (two-tailed)	<0.001		<0.001	<0.001	<0.001	<0.001	<0.001	<0.001	0.112	<0.001	0.612	0.191	0.001	0.612	<0.001	0.015	<0.001	<0.001	0.002
F1_F3	Pearson’s r	0.324 **	0.654 **	1	0.176	0.440 **	0.438 **	0.611 **	0.295 **	0.108	0.360 **	−0.075	0.008	0.277 **	−0.128	0.261 **	0.123	0.341 **	0.332 **	0.011
	Sig. (two-tailed)	<0.001	<0.001		0.054	<0.001	<0.001	<0.001	0.001	0.240	<0.001	0.414	0.930	0.002	0.164	0.004	0.180	<0.001	<0.001	0.905
F1_F4	Pearson’s r	0.243 **	0.303 **	0.176	1	0.544 **	0.465 **	0.340 **	0.583 **	0.440 **	0.401 **	0.402 **	0.582 **	0.262 **	0.360 **	0.396 **	0.348 **	0.313 **	0.510 **	0.404 **
	Sig. (two-tailed)	0.007	<0.001	0.054		<0.001	<0.001	<0.001	<0.001	<0.001	<0.001	<0.001	<0.001	0.004	<0.001	<0.001	<0.001	<0.001	<0.001	<0.001
F1_F5	Pearson’s r	0.451 **	0.534 **	0.440 **	0.544 **	1	0.481 **	0.359 **	0.613 **	0.314 **	0.392 **	0.248 **	0.337 **	0.473 **	0.275 **	0.435 **	0.345 **	0.444 **	0.481 **	0.446 **
	Sig. (two-tailed)	<0.001	<0.001	<0.001	<0.001		<0.001	<0.001	<0.001	<0.001	<0.001	0.006	<0.001	<0.001	0.002	<0.001	<0.001	<0.001	<0.001	<0.001
F1_F6	Pearson’s r	0.128	0.448 **	0.438 **	0.465 **	0.481 **	1	0.532 **	0.346 **	0.198 *	0.331 **	0.106	0.175	0.286 **	0.097	0.313 **	0.249 **	0.458 **	0.371 **	0.133
	Sig. (two-tailed)	0.162	<0.001	<0.001	<0.001	<0.001		<0.001	<0.001	0.030	<0.001	0.248	0.056	0.002	0.293	<0.001	0.006	<0.001	<0.001	0.147
F1_F7	Pearson’s r	0.264 **	0.507 **	0.611 **	0.340 **	0.359 **	0.532 **	1	0.417 **	0.189 *	0.316 **	0.032	0.160	0.252 **	0.012	0.206 *	0.229 *	0.340 **	0.400 **	0.050
	Sig. (two-tailed)	0.004	<0.001	<0.001	<0.001	<0.001	<0.001		<0.001	0.039	<0.001	0.729	0.082	0.005	0.894	0.024	0.012	<0.001	<0.001	0.590
F1_F8	Pearson’s r	0.342 **	0.340 **	0.295 **	0.583 **	0.613 **	0.346 **	0.417 **	1	0.521 **	0.396 **	0.463 **	0.370 **	0.410 **	0.263 **	0.365 **	0.333 **	0.311 **	0.457 **	0.332 **
	Sig. (two-tailed)	<0.001	<0.001	0.001	<0.001	<0.001	<0.001	<0.001		<0.001	<0.001	<0.001	<0.001	<0.001	0.004	<0.001	<0.001	<0.001	<0.001	<0.001
F2_F1	Pearson’s r	0.200 *	0.146	0.108	0.440 **	0.314 **	0.198 *	0.189 *	0.521 **	1	0.500 **	0.664 **	0.573 **	0.497 **	0.506 **	0.238 **	0.343 **	0.167	0.549 **	0.549 **
	Sig. (two-tailed)	0.029	0.112	0.240	<0.001	<0.001	0.030	0.039	<0.001		<0.001	<0.001	<0.001	<0.001	<0.001	0.009	<0.001	0.068	<0.001	<0.001
F2_F2	Pearson’s r	0.293 **	0.383 **	0.360 **	0.401 **	0.392 **	0.331 **	0.316 **	0.396 **	0.500 **	1	0.304 **	0.362 **	0.337 **	0.118	0.421 **	0.378 **	0.391 **	0.583 **	0.264 **
	Sig. (two-tailed)	0.001	<0.001	<0.001	<0.001	<0.001	<0.001	<0.001	<0.001	<0.001		<0.001	<0.001	<0.001	0.199	<0.001	<0.001	<0.001	<0.001	0.004
F2_F3	Pearson’s r	0.145	0.047	−0.075	0.402 **	0.248 **	0.106	0.032	0.463 **	0.664 **	0.304 **	1	0.592 **	0.381 **	0.514 **	0.193 *	0.245 **	0.146	0.445 **	0.461 **
	Sig. (two-tailed)	0.114	0.612	0.414	<0.001	0.006	0.248	0.729	<0.001	<0.001	<0.001		<0.001	<0.001	<0.001	0.035	0.007	0.111	<0.001	<0.001
F2_F4	Pearson’s r	0.150	0.120	0.008	0.582 **	0.337 **	0.175	0.160	0.370 **	0.573 **	0.362 **	0.592 **	1	0.508 **	0.560 **	0.400 **	0.487 **	0.299 **	0.555 **	0.529 **
	Sig. (two-tailed)	0.103	0.191	0.930	<0.001	<0.001	0.056	0.082	<0.001	<0.001	<0.001	<0.001		<0.001	<0.001	<0.001	<0.001	<0.001	<0.001	<0.001
F3_F1	Pearson’s r	0.287 **	0.292 **	0.277 **	0.262 **	0.473 **	0.286 **	0.252 **	0.410 **	0.497 **	0.337 **	0.381 **	0.508 **	1	0.352 **	0.599 **	0.511 **	0.455 **	0.536 **	0.375 **
	Sig. (two-tailed)	0.001	0.001	0.002	0.004	<0.001	0.002	0.005	<0.001	<0.001	<0.001	<0.001	<0.001		<0.001	<0.001	<0.001	<0.001	<0.001	<0.001
F3_F2	Pearson’s r	0.216 *	0.047	−0.128	0.360 **	0.275 **	0.097	0.012	0.263 **	0.506 **	0.118	0.514 **	0.560 **	0.352 **	1	0.178	0.369 **	0.160	0.355 **	0.635 **
	Sig. (two-tailed)	0.018	0.612	0.164	<0.001	0.002	0.293	0.894	0.004	<0.001	0.199	<0.001	<0.001	<0.001		0.052	<0.001	0.081	<0.001	<0.001
F3_F3	Pearson’s r	0.276 **	0.392 **	0.261 **	0.396 **	0.435 **	0.313 **	0.206 *	0.365 **	0.238 **	0.421 **	0.193 *	0.400 **	0.599 **	0.178	1	0.669 **	0.658 **	0.544 **	0.283 **
	Sig. (two-tailed)	0.002	<0.001	0.004	<0.001	<0.001	<0.001	0.024	<0.001	0.009	<0.001	0.035	<0.001	<0.001	0.052		<0.001	<0.001	<0.001	0.002
F3_F4	Pearson’s r	0.193 *	0.221 *	0.123	0.348 **	0.345 **	0.249 **	0.229 *	0.333 **	0.343 **	0.378 **	0.245 **	0.487 **	0.511 **	0.369 **	0.669 **	1	0.624 **	0.620 **	0.414 **
	Sig. (two-tailed)	0.035	0.015	0.180	<0.001	<0.001	0.006	0.012	<0.001	<0.001	<0.001	0.007	<0.001	<0.001	<0.001	<0.001		<0.001	<0.001	<0.001
F3_F5	Pearson’s r	0.199 *	0.498 **	0.341 **	0.313 **	0.444 **	0.458 **	0.340 **	0.311 **	0.167	0.391 **	0.146	0.299 **	0.455 **	0.160	0.658 **	0.624 **	1	0.628 **	0.254 **
	Sig. (two-tailed)	0.030	<0.001	<0.001	<0.001	<0.001	<0.001	<0.001	<0.001	0.068	<0.001	0.111	<0.001	<0.001	0.081	<0.001	<0.001		<0.001	0.005
F3_F6	Pearson’s r	0.302 **	0.460 **	0.332 **	0.510 **	0.481 **	0.371 **	0.400 **	0.457 **	0.549 **	0.583 **	0.445 **	0.555 **	0.536 **	0.355 **	0.544 **	0.620 **	0.628 **	1	0.493 **
	Sig. (two-tailed)	<0.001	<0.001	<0.001	<0.001	<0.001	<0.001	<0.001	<0.001	<0.001	<0.001	<0.001	<0.001	<0.001	<0.001	<0.001	<0.001	<0.001		<0.001
F3_F7	Pearson’s r	0.349 **	0.284 **	0.011	0.404 **	0.446 **	0.133	0.050	0.332 **	0.549 **	0.264 **	0.461 **	0.529 **	0.375 **	0.635 **	0.283 **	0.414 **	0.254 **	0.493 **	1
	Sig. (two-tailed)	<0.001	0.002	0.905	<0.001	<0.001	0.147	0.590	<0.001	<0.001	0.004	<0.001	<0.001	<0.001	<0.001	0.002	<0.001	0.005	<0.001	

** *p* < 0.001; * *p* < 0.05 (two-tailed).
