# Supplementary material for: Competency Goals in Midwifery Master’s Programs in Germany and Selected OECD Countries: Comparison of Stakeholder Perspectives
Source: Healthcare (Basel). 2026 May 18;14(10):1377. doi: 10.3390/healthcare14101377 (PMC13206547; doi:10.3390/healthcare14101377)
Supplement: Supplementary file 1 [file healthcare-14-01377-s001.zip › S1-Informed_consent_English.pdf]

Informed consent for the research project "Assessment of competence goals of master's programmes in midwifery"

Scientific supervision

Prof. Dr. Markus Antonius Wirtz
Freiburg University of Education, Institute for Everyday Culture, Movement and Health, Department of Research Methods, Freiburg, Germany
email: markus.wirtz@ph-freiburg.de

Prof. Dr. Harald Abele, MHBA
University of Tübingen, Institute of Health Sciences, Department of Midwifery Science, Tübingen, Germany
University Hospital Tübingen, Department of Women's Health, Tübingen, Germany
email: Harald.Abele@med.uni-tuebingen.de

Contact person

Angela Kranz, M.Sc.
University of Tübingen, Institute of Health Sciences, Department of Midwifery Science, Tübingen, Germany
email: Angela.Kranz@med.uni-tuebingen.de

Dear participants,

First of all, we would like to thank you for your willingness to participate in this study. In the following, you will be informed about the research project and the data protection. If you agree to participate in the study, you will be asked to create a personal identification number (see below). This is followed by a few details on sociodemographic characteristics before the survey begins. The questionnaire is divided into three superordinate competency areas: General competences, Advanced Midwifery Practice competences and Midwifery Educator competences. Under each of the competency areas you will be asked about the associated competences focusing on your assessment of the importance of these competences in Master's programmes in midwifery.

The terms "woman" and "mother" used in this survey refer to the biological female sex and are independent of a person's gender identity.

(1) Key information on the research project
This research project addresses the frequently demanded necessity of educational research in midwifery and enables implications for the (further) development of Master's programmes in midwifery. The objective is to investigate the expectations on competence goals of midwifery-related Master's programmes in selected OECD countries, involving various actors (students and lecturers in Master's programmes in midwifery, persons working in a professional setting with academic midwives). Potential differences as well as critical insights for improvement will be identified. Additionally, the questionnaire will be subjected to a differentiated statistical examination, thus further developing the survey instrument for educational research in midwifery.
The research project is scientifically accompanied within the framework of a dissertation. Scientific supervision is provided by the Freiburg University of Education, Institute for Everyday Culture, Movement and Health, Department of Research Methods (directed by Prof. Dr. Markus Antonius Wirtz) and by the University of Tübingen, Institute for Health Science, Department of Midwifery Science (directed by Prof. Dr. Harald Abele). The data collected during the research project will be used to answer the relevant research questions of the project. The planned duration of the research project is approximately three years (from the beginning of 2023 to the beginning of 2026), with a limited extension possible. The survey is scheduled from February 2024 to December 2024. The study is financed through funding from the department of Midwifery Science, University of Tübingen as well as through funding for doctoral students of the Freiburg University of Education. Participation is voluntary and can be discontinued at any time. There are no disadvantages for the respondent in the event of non-participation or discontinuation. The duration for completing the survey is about 20 minutes.

Informed consent for the research project "Assessment of competence goals of master's programmes in midwifery"

(2) Responsibility for data collection and contact persons

The study supervision (Prof. Dr. Harald Abele) is responsible for compliance with data protection.

Study supervision

Prof. Dr. Harald Abele

University of Tübingen, Institute of Health Sciences, Department of Midwifery, Hoppe-Seyler-Straße 9, 72076 Tübingen, Germany

University Hospital Tübingen, Department of Women's Health, Calwerstraße 7, 72076 Tübingen

Study support

Angela Kranz, M.Sc. Health Pedagogy

University of Tübingen, Institute of Health Science, Department of Midwifery, Hoppe-Seyler-Straße 9, 72076 Tübingen, Germany

email: Angela.Kranz@med.uni-tuebingen.de

phone: +49 7071 29-87447

Contact details of the data protection office

Data Protection Officer University of Tübingen

Geschwister-Scholl-Platz, 72074 Tübingen, Germany

email: Datenschutz@uni-tuebingen.de

phone: +49 70 71 29-0

(3) Collection of personal and non-personal data

In the context of the present research project, the following personal data will be collected according to art. 4, no. 1 GDPR (General Data Protection Regulation): Gender (female, male, diverse): Age in years; Country of work or study; Professional degree (related to midwifery science; nursing science or equivalent); Professional degree parents (minimum Bachelor's degree or higher; Yes/No query); Belonging to study population (students and lecturers in Master's programmes in midwifery, persons working in a professional setting with academic midwives). All other data collected are non-personal data (information on assessments of competency goals in midwifery-related master's programmes). Nevertheless, the GDPR is applied to the entire data set, as they are inextricably linked to personal data. The legal basis for processing personal data is consent according to art. 6, no. 1 a GDPR, which appears at the end of the clarification. The data is collected to meet the study objectives. Aspects are to be identified that influence expectations about competence goals in midwifery-related Master's programs. For this purpose, differentiated statistical evaluations are necessary through the described person-related and non-person-related data. Thus, the data is collected for scientific purposes according to §13 Landesdatenschutzgesetz Baden-Württemberg (LSDG BW).

(4) Data evaluation

In accordance with art. 4, no. 5 GDPR, data is collected and analysed in pseudonymized form using a personal identification number. This is created by the participants themselves on the basis of instructions (after consent to participate in the survey is given, the instructions for creating a personal identification number appear). Only the participants themselves are aware of this identification number. The data is therefore protected and do not allow any conclusions to be drawn about a person. The identification number allows the recognition of the corresponding data record when exercising the right object or delete data as stated in Article 17 and Article 21 of the GDPR. This means that third parties, including the study staff, cannot identify the participants through the survey data.

The data are analysed and used exclusively for scientific purposes (by authorized study staff using IBM SPSS 26 statistical software). The data will be published in pseudonymised, statistically aggregated and summarised form in scientific publications within professional journals. Identification numbers are removed during the analysis and presentation of the results.

(5) Data storage

The online survey provider *LimeSurvey* initially stores the collected data in encrypted form on their (German) servers. Here, the encrypted data is stored in a separate database. It is not possible to infer the time of survey participation or the IP address of the participants. An agreement on commissioned processing has been drawn up with LimeSurvey in accordance with art. 28 GDPR. LimeSurvey is compatible with the GDPR and EU law.

Scientific supervision
Prof. Dr. Markus Antonius Wirtz
email: markus.wirtz@ph-freiburg.de
Prof. Dr. Harald Abele, MHBA
email: Harald.Abele@med.uni-tuebingen.de

Contact person
Angela Kranz, M.Sc.
email: Angela.Kranz@med.uni-tuebingen.de

After the survey phase ends, the data is exported to internal servers of the University of Tübingen, to which only authorized scientists have access. This is password-protected. The necessary access control is also ensured by appropriate building and room security. Following the export of the data to internal servers of the University of Tübingen, the irrevocable deletion of the data on LimeSurvey is initiated. After the pseudonymised data has been processed for research purposes, it is stored electronically for 10 years according to the recommendation of the German Research Foundation. After this period, the data will be finally and irrevocably deleted. The information obtained from the survey data is processed exclusively in the EU, the European Economic Area or in countries with comparable levels of data protection.

(6) The rights of the study participants
According to art. 21 GDPR, consent under data protection law can be revoked at any time. In addition, the further processing of the data can be obtained, from which evaluations already carried out remain unaffected (art. 7 GDPR). It is possible at any time to obtain information about the stored personal data (art. 15 GDPR), to delete the data (art. 17 GDPR) or to entitle (art. 16 GDPR). Furthermore, the claim of restriction of data processing is possible at any time (art. 18 GDPR), as well as the right to data portability (art. 20 GDPR).
There are no disadvantages for the data subject as a result of revocation or non-participation. If you wish to exercise these rights, please contact the responsible contact person.

Angela Kranz, M.Sc. Health Pedagogy
University of Tübingen, Institute of Health Sciences, Department of Midwifery, Hoppe-Seyler-Straße 9, 72076 Tübingen, Germany
email: Angela.Kranz@med.uni-tuebingen.de
phone: +49 7071 29-87447

One also has the right to complain to the competent supervisory authority for data protection.
State Commissioner for Data Protection and Freedom of Information in Baden-Württemberg,
P.O. Box 10 29 32 70025 Stuttgart
email: Poststelle@lfdi.bwl.de
phone: 0711 / 61 55 41 - 716

The legal basis for processing the aforementioned personal data is the consent, pursuant to art. 6, no. 1 a GDPR, obtained from the information provided here.

Consent *[only possible online via the survey link]*
