# Supplementary material for: Competency Goals in Midwifery Master’s Programs in Germany and Selected OECD Countries: Comparison of Stakeholder Perspectives
Source: Healthcare (Basel). 2026 May 18;14(10):1377. doi: 10.3390/healthcare14101377 (PMC13206547; doi:10.3390/healthcare14101377)
Supplement: Supplementary file 1 [file healthcare-14-01377-s001.zip › S2-Informed_consent_German.pdf]

Aufklärungstext und Einverständniserklärung für die Studienteilnehmende zum Forschungsprojekt „Kompetenzziele in hebammenbezogenen Masterstudiengängen“

Sehr geehrte Teilnehmende,

Zunächst möchten wir uns herzlich für Ihre Bereitschaft an der Studie teilzunehmen bedanken. Im Folgenden werden Sie über das Forschungsvorhaben und die datenschutzrechtlichen Aspekte aufgeklärt. Insofern Sie mit der Teilnahme an der Studie einverstanden sind, werden Sie zunächst gebeten, eine persönliche Identifikationsnummer zu erstellen (s.u.). Anschließend folgen wenige Angaben zu soziodemographischen Merkmalen, bevor schließlich die Befragung beginnt. Der Fragebogen ist in drei übergeordnete Kompetenzbereiche gegliedert: Allgemeine Kompetenzen, Advanced Midwifery Practice Kompetenzen sowie Hebammen Lehrende/-r Kompetenzen. Unter jedem übergeordneten Kompetenzbereich werden Sie nach jeweiligen dazugehörigen Kompetenzen gefragt. Hierbei dürfen Sie die Wichtigkeit der Kompetenzen in Masterstudiengängen im Hebammenwesen einschätzen.

Die in dieser Befragung verwendeten Begriffe „Frau“ und „Mutter“ beziehen sich auf das biologische weibliche Geschlecht und sind unabhängig von der Genderidentität einer Person.

(1) Eckdaten zum Forschungsvorhaben

Das Forschungsvorhaben kommt der vielfach geforderten Notwendigkeit von Bildungsforschung im Hebammenwesen nach und ermöglicht hierdurch praktische Implikationen für die (Weiter-)Entwicklung von hebammenbezogenen Masterstudiengängen. Das Ziel liegt darin, die Erwartungen zu den Kompetenzzielen nach Absolvierung von hebammenbezogenen Masterstudiengängen in ausgewählten OECD-Ländern unter Einbezug verschiedener Akteurinnen und Akteure (Studierende/-r, Dozierende/-r in hebammenbezogenen Masterstudiengängen oder Personen, die in einem professionellen Umfeld mit akademisierten Hebammen zusammenarbeiten) zu untersuchen. Hieraus sollen mögliche Differenzen sowie entscheidende Verbesserungshinweise identifiziert werden. Darüber hinaus wird der hier vorgelegte Fragebogen einer differenzierten statistischen Prüfung unterzogen, sodass als ein weiteres Ziel das Erhebungsinstrument für die Bildungsforschung im Hebammenwesen (weiter-)entwickelt wird.
Das **Forschungsvorhaben wird wissenschaftlich im Rahmen einer Dissertation begleitet**. Die wissenschaftliche Betreuung erfolgt durch die Pädagogische Hochschule Freiburg, Institut für Alltagskultur, Bewegung und Gesundheit, Fachrichtung Forschungsmethoden (Leitung Prof. Dr. Markus Antonius Wirtz) sowie durch die Universität Tübingen, Institut für Gesundheitswissenschaft, Abteilung Hebammenwissenschaft (Leitung Prof. Dr. Harald Abele). Die im Rahmen des Forschungsvorhabens erhobenen Daten werden zur Beantwortung der relevanten Forschungsfragen des Vorhabens herangezogen. Die vorgesehene Laufzeit des Forschungsvorhabens beträgt ca. drei Jahre (von Anfang 2023 bis Anfang 2026), wobei eine begrenzte Verlängerung möglich ist. Die Befragung ist von Februar 2024 bis Dezember 2024 vorgesehen. Die Studie wird durch Eigenmittel der Abteilung Hebammenwissenschaft, Universität Tübingen sowie durch Fördermittel für Doktorand/-innen der Pädagogischen Hochschule Freiburg finanziert. Die Teilnahme an der Befragung ist freiwillig und kann jederzeit abgebrochen werden.

Wissenschaftliche Betreuung

Prof. Dr. Markus Antonius Wirtz
Pädagogische Hochschule Freiburg, Institut für Alltagskultur, Bewegung und Gesundheit, Fachrichtung Forschungsmethoden, Freiburg, Deutschland
E-Mail: markus.wirtz@ph-freiburg.de

Prof. Dr. Harald Abele, MHBA
Universität Tübingen, Institut für Gesundheitswissenschaft, Abteilung für Hebammenwissenschaft, Tübingen, Deutschland
Universitätsklinikum Tübingen, Tübingen, Deutschland
E-Mail: Harald.Abele@med.uni-tuebingen.de

Ansprechpartnerin

Angela Kranz, M.Sc.
Universität Tübingen, Institut für Gesundheitswissenschaft, Abteilung für Hebammenwissenschaft, Tübingen, Deutschland
E-Mail: Angela.Kranz@med.uni-tuebingen.de

Aufklärungstext und Einverständniserklärung für die Studienteilnehmende zum Forschungsprojekt „Kompetenzziele in hebammenbezogenen Masterstudiengängen“

Bei Nichtteilnahme oder Abbruch entstehen für die befragte Person keinerlei Nachteile. Die Bearbeitungsdauer für die Befragung beträgt ca. 20 Minuten.

(2) Verantwortung der Datenerhebung sowie Ansprechpartner/-innen
Der Studienleitung (Prof. Dr. Harald Abele) obliegt die Verantwortung zur Einhaltung des Datenschutzes.
Studienleitung
Prof. Dr. Harald Abele
Universität Tübingen, Institut für Gesundheitswissenschaft, Abteilung für Hebammenwissenschaft, Hoppe-Seyler-Straße 9, 72076 Tübingen
Universitätsklinikum Tübingen, Department für Frauengesundheit, Calwerstraße 7, 72076 Tübingen
Studienbetreuung
Angela Kranz, M.Sc. Gesundheitspädagogik
Universität Tübingen, Institut für Gesundheitswissenschaft, Abteilung für Hebammenwissenschaft, Hoppe-Seyler-Straße 9, 72076 Tübingen
E-Mail: Angela.Kranz@med.uni-tuebingen.de
Telefon: +49 7071 29-87447
Kontaktdaten des Datenschutzbeauftragten
Datenschutzbeauftragter Universität Tübingen
Geschwister-Scholl-Platz, 72074 Tübingen
E-Mail: Datenschutz@uni-tuebingen.de
Telefon: +49 70 71 29-0

(3) Erhebung personenbezogener und nicht personenbezogener Daten
Im Rahmen des vorliegenden Forschungsvorhabens werden nach Art. 4, Nr. 1 DSGVO (Datenschutzgrundverordnung) folgende personenbezogenen Daten erhoben: Geschlechtszugehörigkeit (Weiblich, Männlich, Divers): Alter in Jahren; Land, in dem gearbeitet oder studiert wird; beruflicher Abschluss (bezogen auf Hebammenwissenschaft; Pflegewissenschaft oder Vergleichbares); beruflicher Abschluss Eltern (mindestens Bachelorabschluss oder höher; Ja/Nein Abfrage); Zugehörigkeit zu Studienpopulation (Studierende/-r, Dozierende/-r in hebammenbezogenen Masterstudiengängen oder Personen, die in einem professionellem Umfeld mit akademisierten Hebammen zusammenarbeiten). Aller weiteren Daten, die erhoben werden, sind nicht personenbezogene Daten (Informationen zu Einschätzungen der Kompetenzziele in hebammenbezogenen Masterstudiengängen). Die DSGVO wird dennoch auf den gesamten Datensatz übertragen, da diese untrennbar mit personenbezogenen Daten verbunden sind. Die Rechtsgrundlage zur Verarbeitung personenbezogener Daten bildet die Einwilligung nach Art. 6, Abs. 1 lit. a DSGVO, die am Ende der Aufklärung erscheint.
Die Daten werden erhoben, um den Studienzielen gerecht zu werden. Es sollen Aspekte identifiziert werden, die auf die Erwartungen zu Kompetenzzielen in hebammenbezogenen Masterstudiengängen Einfluss nehmen. Hierfür sind differenzierte statistische Auswertungen durch die beschriebenen personenbezogenen und nicht personenbezogenen Daten notwendig. Damit werden die Daten zu wissenschaftlichen Zwecken nach §13 Landesdatenschutzgesetz Baden-Württemberg (LSDG BW) erhoben.

(4) Datenauswertung
Die Erhebung sowie Auswertung der Daten erfolgt nach Art. 4, Abs. 5 DSGVO in pseudonymisierter Form unter der Verwendung einer persönlichen Identifikationsnummer. Diese wird anhand einer Anleitung von den Teilnehmenden selbst erstellt (nachdem die Einwilligung zur Teilnahme an der Befragung erfolgt, erscheint die Anleitung zur Erstellung einer persönlichen Identifikationsnummer). Allein die Teilnehmenden selbst sind in Kenntnis über diese Identifikationsnummer. Die Daten sind somit geschützt und lassen keinen Rückschluss auf eine Person zu. Insofern von dem Recht des Widerspruchs oder Löschung der Daten nach Art. 17 und Art. 21 DSGVO Gebrauch gemacht wird, ermöglicht die Identifikationsnummer eine Erkennung des entsprechenden Datensatzes. Dritten (auch den Studienmitarbeitenden) ist eine Identifikation der Teilnehmenden mittels der Erhebungsdaten somit unmöglich. Die Daten werden ausschließlich zu wissenschaftlichen Zwecken ausgewertet (durch befugte Studienmitarbeitende mittels der Statistiksoftware IBM SPSS 29) und verwendet. Die Veröffentlichung der Daten erfolgt in pseudonymisierter,

statistisch aggregierter und zusammengefasster Form in wissenschaftlichen Publikationen in Fachzeitschriften. Die Identifikationsnummern werden bei der Ergebnisauswertung und -darstellung entfernt.

(5) Datenspeicherung
Der Anbieter für Online-Umfragen *LimeSurvey* speichert die erhobenen Daten zunächst verschlüsselt auf deren (deutschen) Servern. Hier sind die verschlüsselten Daten in einer separaten Datenbank abgespeichert. Es ist kein Rückschluss auf den Zeitpunkt der Umfrageteilnahme sowie die IP-Adresse der Teilnehmenden möglich. Mit LimeSurvey wurde gemäß Art. 28 DSGVO eine Vereinbarung zur Auftragsverarbeitung erstellt. LimeSurvey ist mit der DSGVO und dem EU-Recht kompatibel.
Nachdem die Erhebungsphase endet, werden die Daten auf interne Server der Universität Tübingen exportiert, auf welche nur befugte Wissenschaftler/-innen Zugriff haben. Dies erfolgt passwortgeschützt. Die notwendige Zutrittskontrolle ist durch eine angemessene Gebäude- und Raumsicherung ebenfalls gewährleistet. Im Anschluss an den Export der Daten auf interne Server der Universität Tübingen, wird die unwiderrufliche Löschung der Daten auf LimeSurvey eingeleitet. Nachdem die pseudonymisierten Daten zu Forschungszwecken verarbeitet wurden, werden diese für 10 Jahre gemäß der Empfehlung der Deutschen Forschungsgemeinschaft elektronisch abgespeichert. Nach dieser Frist werden die Daten schließlich endgültig und unwiderruflich gelöscht. Die gewonnen Informationen anhand der Erhebungsdaten werden ausschließlich in der EU, dem Europäischen Wirtschaftsraum oder in Ländern mit vergleichbaren Datenschutzniveau verarbeitet.

(6) Die Rechte der Studienteilnehmenden
Nach Art. 21 DSGVO kann die datenschutzrechtliche Einwilligung jederzeit widerrufen werden. Darüber hinaus kann die weitere Verarbeitung der Daten erwirkt werden, wovon bereits durchgeführte Auswertungen unberührt bleiben (Art. 7 DSGVO). Es besteht jederzeit die Möglichkeit Auskunft über die gespeicherten personenbezogenen Daten zu erhalten (Art. 15 DSGVO), die Daten zu löschen (Art. 17 DSGVO) oder zu berechtigen (Art. 16 DSGVO). Des Weiteren ist die Beanspruchung der Einschränkung der Datenverarbeitung jederzeit möglich (Art. 18 DSGVO), ebenso wie das Recht auf Datenübertragbarkeit (Art. 20 DSGVO). Es entstehen durch den Widerruf oder durch eine Nichtteilnahme keinerlei Nachteile für die betroffene Person.

Insofern diese Rechte in Anspruch genommen werden wollen, dann kann sich an die verantwortliche Ansprechpartnerin gewendet werden.

Angela Kranz, M.Sc. Gesundheitspädagogik
Universität Tübingen, Institut für Gesundheitswissenschaft, Abteilung für Hebammenwissenschaft, Hoppe-Seyler-Straße 9, 72076 Tübingen
E-Mail: Angela.Kranz@med.uni-tuebingen.de
Telefon: +49 7071 29-87447

Außerdem besteht das Recht sich bei der zuständigen Aufsichtsbehörde für den Datenschutz zu beschweren.

Landesbeauftragter für den Datenschutz und die Informationsfreiheit in Baden-Württemberg,
Postfach 10 29 32
70025 Stuttgart
E-Mail: Poststelle@lfdi.bwl.de
Telefon: +49 711 / 61 55 41 – 716

Die Rechtsgrundlage zur Verarbeitung der genannten personenbezogenen Daten bildet die Einwilligung gemäß Art. 6, Abs. 1 lit. a DSGVO von den hier zur Verfügung gestellten Informationen.

Zustimmung *[ist nur online über den Befragungslink möglich]*

Wissenschaftliche Betreuung
Prof. Dr. Markus Antonius Wirtz
E-Mail: markus.wirtz@ph-freiburg.de
Prof. Dr. Harald Abele, MHBA
E-Mail: Harald.Abele@med.uni-tuebingen.de

Ansprechpartnerin
Angela Kranz, M.Sc.
E-Mail: Angela.Kranz@med.uni-tuebingen.de
