# Supplementary material for: Competency Goals in Midwifery Master’s Programs in Germany and Selected OECD Countries: Comparison of Stakeholder Perspectives
Source: Healthcare (Basel). 2026 May 18;14(10):1377. doi: 10.3390/healthcare14101377 (PMC13206547; doi:10.3390/healthcare14101377)
Supplement: Supplementary file 1 [file healthcare-14-01377-s001.zip › S3-Survey_Instrument_English.pdf]

Section A: Personal identification number

A1.

Please create a personal identification number. This serves two primary purposes: (1) To identify the specific data set in case you claim your rights, as previously described, either to object to or delete the data set. (2) It serves to recognise whether participants have accidentally taken part twice in the survey.

It is not possible to draw conclusions about your person. Please keep the personal identification number in a safe place. If you do not know any of the above information, please enter an X. Example: Cologne, Bernd, December, 16.09.1996 [illegible] CBDC16

The personal identification number consists of the following information:

1. The first letter of your place of birth

A2. 2. The first letter of your father's first name

A3. 3. The last letter of your father's first name

A4. **4. The third letter of your birth month**

A5. **5. The first two digits of your date of birth (DD.MM.YYYY)**

Section B: 12. Sociodemographic characteristics

B1. **Which gender do you belong to?**

- Female ☐
- Male ☐
- Diverse ☐

B2. **How old are you (in years)?**

B3. **In which country do you currently work or study in?**

B4. **What is your professional degree?**

- Completed vocational training in midwifery ☐
- Completed vocational training in nursing ☐

Completed Bachelor's degree (or equivalent) in midwifery science ☐

Completed Master's degree (or equivalent) in midwifery science ☐

Completed Bachelor's degree (or equivalent) in nursing science ☐

Completed Master's degree (or equivalent) in nursing science ☐

Completed Bachelor’s degree in related profession ☐

Completed Master’s degree in related profession ☐

Doctorate ☐

Habilitation ☐

Other professional degree ☐

B5. Have your parents (at least one parent) completed higher education? (Bachelor's degree / equivalent or higher)

Yes ☐

No ☐

B6. Which of the following designation applies to you?

Student in Master's programme in midwifery science ☐

Lecturer in Master's programme midwifery science ☐

Working in a professional environment outside of higher education related to midwifery ☐

Lecturer in Bachelor's programme midwifery science ☐

Section C: General competences: Ability to promote evidence-based practice

Section I: General competences

C1. When students start the Master’s programme, they already have acquired basic competences of midwifery practice.

To what extent is it important that the following competences are developed or deepened in the Master’s programme?

	1 Not important in the Master’s programme [Competences sufficient through previous qualification]	2 Moderately important in the Master’s programme	3 Rather important in the Master’s programme	4 Important in the Master’s programme	5 Very important in the Master’s programme
I-1-1 Ability to identify gaps between evidence and practice	☐	☐	☐	☐	☐
I-1-2 Ability to find solutions for bridging the gaps between evidence and practice	☐	☐	☐	☐	☐
I-1-3 Ability to integrate research findings from midwifery and related sciences into processes of evidence-based decision-making	☐	☐	☐	☐	☐
I-1-4 Ability to actively promote evidence-based midwifery practice	☐	☐	☐	☐	☐

	1 Not important in the Master's programme [Competences sufficient through previous qualification]	2 Moderately important in the Master's programme	3 Rather important in the Master's programme	4 Important in the Master's programme	5 Very important in the Master's programme
I-1-5 Ability to assess and utilise research findings to facilitate evidence-based health care	☐	☐	☐	☐	☐

Section D: General competences: Ability to initiate and coordinate midwifery research and to evaluate and apply best-practice models

Section I: General competences

D1. When students start the Master's programme, they already have acquired basic competences of midwifery practice.

To what extent is it important that the following competences are developed or deepened in the Master's programme?

	1 Not important in the Master's programme [Competences sufficient through previous qualification]	2 Moderately important in the Master's programme	3 Rather important in the Master's programme	4 Important in the Master's programme	5 Very important in the Master's programme
I-2-1 Ability to evaluate and use research findings for initiating improvements in midwifery practice	☐	☐	☐	☐	☐
I-2-2 Ability to initiate and coordinate research for advancing high-quality health care	☐	☐	☐	☐	☐
I-2-3 Ability to develop concepts and models of care based on research findings from midwifery and related sciences	☐	☐	☐	☐	☐

Section E: General competences: Ability to initiate and coordinate midwifery research and to evaluate and apply best-practice models

Section I: General competences

E1. When students start the Master's programme, they already have acquired basic competences of midwifery practice.

To what extent is it important that the following competences are developed or deepened in the Master's programme?

	1 Not important in the Master's programme [Competences sufficient through previous qualification]	2 Moderately important in the Master's programme	3 Rather important in the Master's programme	4 Important in the Master's programme	5 Very important in the Master's programme
I-2-3 Ability to evaluate and apply clinical expertise into best practice-models	☐	☐	☐	☐	☐
I-2-5 Ability to use relevant data for analysing outcomes in midwifery practice	☐	☐	☐	☐	☐
I-2-6 Ability to connect relevant data to best-practice models	☐	☐	☐	☐	☐

Section F: General competences: Ability to analyse health policy

Section I: General competences

F1. When students start the Master's programme, they already have acquired basic competences of midwifery practice.

To what extent is it important that the following competences are developed or deepened in the Master's programme?

	1 Not important in the Master's programme [Competences sufficient through previous qualification]	2 Moderately important in the Master's programme	3 Rather important in the Master's programme	4 Important in the Master's programme	5 Very important in the Master's programme
I-3-1 Ability to analyse the process of health policy development	☐	☐	☐	☐	☐
I-3-2 Ability to analyse influential factors on health policy	☐	☐	☐	☐	☐
I-3-3 Ability to analyse policy effects on clinical practice	☐	☐	☐	☐	☐

Section G: General competences: Ability to work in interprofessional collaboration

Section I: General competences

G1. When students start the Master's programme, they already have acquired basic competences of midwifery practice.

To what extent is it important that the following competences are developed or deepened in the Master's programme?

	1 Not important in the Master's programme [Competences sufficient through previous qualification]	2 Moderately important in the Master's programme	3 Rather important in the Master's programme	4 Important in the Master's programme	5 Very important in the Master's programme
I-4-1 Ability to work as an effective team member in interprofessional collaboration	☐	☐	☐	☐	☐
I-4-2 Ability to improve interprofessional collaboration for strengthening positive change in health care	☐	☐	☐	☐	☐

Section H: General competences: Ability to identify midwifery theories

Section I: General competences

H1. When students start the Master's programme, they already have acquired basic competences of midwifery practice.

To what extent is it important that the following competences are developed or deepened in the Master's programme?

	1 Not important in the Master's programme [Competences sufficient through previous qualification]	2 Moderately important in the Master's programme	3 Rather important in the Master's programme	4 Important in the Master's programme	5 Very important in the Master's programme
I-5-1 Ability to identify relevant theories to midwifery practice	☐	☐	☐	☐	☐
I-5-2 Ability to identify relevant theories to midwifery science	☐	☐	☐	☐	☐
I-5-3 Ability to identify relevant theories to the promotion of health equity and social justice	☐	☐	☐	☐	☐
I-5-4 Ability to identify relevant theories to ethical principles in midwifery care	☐	☐	☐	☐	☐

Section I: General competences: Ability to use technologies

Section I: General competences

I1. When students start the Master's programme, they already have acquired basic competences of midwifery practice.

To what extent is it important that the following competences are developed or deepened in the Master's programme?

	1 Not important in the Master's programme [Competences sufficient through previous qualification]	2 Moderately important in the Master's programme	3 Rather important in the Master's programme	4 Important in the Master's programme	5 Very important in the Master's programme
I-6-1 Ability to improve the quality of health care practice using technologies	☐	☐	☐	☐	☐
I-6-2 Ability to improve the safety of health care practice using technologies	☐	☐	☐	☐	☐

Section J: General competences: Ability to manage finances and resources

Section I: General competences

J1. **When students start the Master's programme, they already have acquired basic competences of midwifery practice.**

To what extent is it important that the following competences are developed or deepened in the Master's programme?

	1 Not important in the Master's programme [Competences sufficient through previous qualification]	2 Moderately important in the Master's programme	3 Rather important in the Master's programme	4 Important in the Master's programme	5 Very important in the Master's programme
I-7-1 Ability to evaluate financial aspects of health care practice	☐	☐	☐	☐	☐
I-7-2 Ability to use appropriate resources in health care practice	☐	☐	☐	☐	☐
I-7-3 Ability to manage the resources of health care practice	☐	☐	☐	☐	☐

Section K: General competences: Ability to demonstrate the professional understanding of the midwifery profession in different contexts

Section I: General competences

K1. **When students start the Master's programme, they already have acquired basic competences of midwifery practice.**

To what extent is it important that the following competences are developed or deepened in the Master's programme?

	1 Not important in the Master's programme [Competences sufficient through previous qualification]	2 Moderately important in the Master's programme	3 Rather important in the Master's programme	4 Important in the Master's programme	5 Very important in the Master's programme
I-8-1 Ability to demonstrate the professional understanding of the midwifery profession in clinical practice	☐	☐	☐	☐	☐
I-8-2 Ability to demonstrate professional understanding of the midwifery profession in administration	☐	☐	☐	☐	☐
I-8-3 Ability to demonstrate the professional understanding of the midwifery profession in policy implementation	☐	☐	☐	☐	☐
I-8-4 Ability to demonstrate health disparities in midwifery practice	☐	☐	☐	☐	☐
I-8-5 Ability to explore potential areas of interest in midwifery	☐	☐	☐	☐	☐

Section L: General abilities

Section I: General competences

L1. When students start the Master's programme, they already have acquired basic competences of midwifery practice.

To what extent is it important that the following competences are developed or deepened in the Master's programme?

	1 Not important in the Master's programme [Competences sufficient through previous qualification]	2 Moderately important in the Master's programme	3 Rather important in the Master's programme	4 Important in the Master's programme	5 Very important in the Master's programme
C-1 Ability to uphold fundamental human rights of individuals when providing midwifery care	☐	☐	☐	☐	☐
C-2 Ability to adhere jurisdictional laws, regulatory requirements, and codes of conduct for midwifery practice	☐	☐	☐	☐	☐
C-3 Ability to facilitate normal birth processes in institutional and community settings	☐	☐	☐	☐	☐
C-4 Ability to recognise conditions outside midwifery scope of practice and refer appropriately	☐	☐	☐	☐	☐

Section M: Advanced Midwifery Practice competences: Ability to respect the dignity, trust and discretion

Section II: Advanced Midwifery Practice competences

M1. When students start the Master's programme, they already have acquired basic competences of midwifery practice.

To what extent is it important that the following competences are developed or deepened in the Master's programme?

	1 Not important in the Master's programme [Competences sufficient through previous qualification]	2 Moderately important in the Master's programme	3 Rather important in the Master's programme	4 Important in the Master's programme	5 Very important in the Master's programme
II-1-1 Ability to apply ethically sound solutions to complex issues related to the care of women and their babies	☐	☐	☐	☐	☐
II-1-2 Ability to negotiate and advocate the rights and wishes of women, their babies and families with other health professionals and services	☐	☐	☐	☐	☐

LimeSurvey

Section N: Advanced Midwifery Practice competences: Ability to assume professional responsibility and accountability in a leadership role

Section II: Advanced Midwifery Practice competences

N1. When students start the Master's programme, they already have acquired basic competences of midwifery practice.

To what extent is it important that the following competences are developed or deepened in the Master's programme?

	1 Not important in the Master's programme [Competences sufficient through previous qualification]	2 Moderately important in the Master's programme	3 Rather important in the Master's programme	4 Important in the Master's programme	5 Very important in the Master's programme
II-2-1 Ability to use advanced knowledge, skills and abilities for engaging in a leadership role in complex clinical decision-making	☐	☐	☐	☐	☐
II-2-2 Ability to use advanced knowledge, skills and abilities within a leadership role to critically analyse and solve problems	☐	☐	☐	☐	☐

Section O: Advanced Midwifery Practice competences: Ability to protect and promote quality of practice

Section II: Advanced Midwifery Practice competences

O1. When students start the Master's programme, they already have acquired basic competences of midwifery practice.

To what extent is it important that the following competences are developed or deepened in the Master's programme?

	1 Not important in the Master's programme [Competences sufficient through previous qualification]	2 Moderately important in the Master's programme	3 Rather important in the Master's programme	4 Important in the Master's programme	5 Very important in the Master's programme
II-3-1 Ability to promote and protect high-quality maternity care and services	☐	☐	☐	☐	☐
II-3-2 Ability to promote and protect compassionate maternity care and services	☐	☐	☐	☐	☐
II-3-3 Ability to promote and protect evidence-based safe maternity care and services	☐	☐	☐	☐	☐

Section P: Advanced Midwifery Practice competences: Ability for collaborative risk assessment

Section II: Advanced Midwifery Practice competences

P1. **When students start the Master's programme, they already have acquired basic competences of midwifery practice.**

To what extent is it important that the following competences are developed or deepened in the Master's programme?

	1 Not important in the Master's programme [Competences sufficient through previous qualification]	2 Moderately important in the Master's programme	3 Rather important in the Master's programme	4 Important in the Master's programme	5 Very important in the Master's programme
II-4-1 Ability to manage collaborative risk assessment with others	☐	☐	☐	☐	☐
II-4-2 Ability to promote a safe environment in collaboration with others	☐	☐	☐	☐	☐

Section Q: Midwifery Educator competences: Ability to incorporate and promote ethical and legal principles into teaching

Section III: Midwifery Educator competences

Q1. **When students start the Master's programme, they already have acquired basic competences of midwifery practice.**

To what extent is it important that the following competences are developed or deepened in the Master's programme?

	1 Not important in the Master's programme [Competences sufficient through previous qualification]	2 Moderately important in the Master's programme	3 Rather important in the Master's programme	4 Important in the Master's programme	5 Very important in the Master's programme
III-1-1 Ability to integrate ethical aspects of midwifery care into teaching/learning activity	☐	☐	☐	☐	☐
III-1-2 Ability to integrate legal aspects of midwifery care into teaching/learning activity	☐	☐	☐	☐	☐
III-1-3 Ability to promote consistent role modelling of ethical and legal aspects of midwifery care into teaching/learning activity	☐	☐	☐	☐	☐

Section R: Midwifery Educator competences: Ability to maintain and update midwifery competencies in theory and practice

Section III: Midwifery Educator competences

R1. **When students start the Master's programme, they already have acquired basic competences of midwifery practice.**

To what extent is it important that the following competences are developed or deepened in the Master's programme?

	1 Not important in the Master's programme [Competences sufficient through previous qualification]	2 Moderately important in the Master's programme	3 Rather important in the Master's programme	4 Important in the Master's programme	5 Very important in the Master's programme
III-2-1 Ability to maintain knowledge and skills of theory and practice in midwifery up to date	☐	☐	☐	☐	☐

	1 Not important in the Master's programme [Competences sufficient through previous qualification]	2 Moderately important in the Master's programme	3 Rather important in the Master's programme	4 Important in the Master's programme	5 Very important in the Master's programme
III-2-2 Ability to base knowledge and skills of theory and practice in midwifery on the best available evidence	☐	☐	☐	☐	☐

Section S: Midwifery Educator competences: Ability to create a conductive environment for theoretical learning

Section III: Midwifery Educator competences

S1. When students start the Master's programme, they already have acquired basic competences of midwifery practice.

To what extent is it important that the following competences are developed or deepened in the Master's programme?

	1 Not important in the Master's programme [Competences sufficient through previous qualification]	2 Moderately important in the Master's programme	3 Rather important in the Master's programme	4 Important in the Master's programme	5 Very important in the Master's programme
III-3-1 Ability to incorporate educational strategies for promoting active learning	☐	☐	☐	☐	☐
III-3-2 Ability to select and use effective teaching and learning materials	☐	☐	☐	☐	☐
III-3-3 Ability to recognise and support different learning styles and individual learning needs	☐	☐	☐	☐	☐

Section T: Midwifery Educator competences: Ability to create an effective learning environment for clinical teaching

Section III: Midwifery Educator competences

T1. When students start the Master's programme, they already have acquired basic competences of midwifery practice.

To what extent is it important that the following competences are developed or deepened in the Master's programme?

	1 Not important in the Master's programme [Competences sufficient through previous qualification]	2 Moderately important in the Master's programme	3 Rather important in the Master's programme	4 Important in the Master's programme	5 Very important in the Master's programme
III-4-1 Ability to create a safe and effective learning environment in the clinical setting of midwifery care	☐	☐	☐	☐	☐
III-4-2 Ability to promote individual experiential learning	☐	☐	☐	☐	☐

Section U: Midwifery Educator competences: Ability for quality assurance in educational programs

Section III: Midwifery Educator competences

U1. **When students start the Master's programme, they already have acquired basic competences of midwifery practice.**

To what extent is it important that the following competences are developed or deepened in the Master's programme?

	1 Not important in the Master's programme [Competences sufficient through previous qualification]	2 Moderately important in the Master's programme	3 Rather important in the Master's programme	4 Important in the Master's programme	5 Very important in the Master's programme
III-5-1 Ability to regular monitor, assess and evaluate a midwifery education programme	☐	☐	☐	☐	☐
III-5-2 Ability to regular assess the students' competencies	☐	☐	☐	☐	☐

Section V: Midwifery Educator competences: Ability for quality assurance in educational programs

Section III: Midwifery Educator competences

V1. **When students start the Master's programme, they already have acquired basic competences of midwifery practice.**

To what extent is it important that the following competences are developed or deepened in the Master's programme?

	1 Not important in the Master's programme [Competences sufficient through previous qualification]	2 Moderately important in the Master's programme	3 Rather important in the Master's programme	4 Important in the Master's programme	5 Very important in the Master's programme
III-5-3 Ability to actively participate in the organisation and implementation of a midwifery curriculum	☐	☐	☐	☐	☐
III-5-4 Ability to revise and implement a midwifery education programme	☐	☐	☐	☐	☐

Section W: Midwifery Educator competences: Ability to advocate and act as a leader

Section III: Midwifery Educator competences

W1. When students start the Master's programme, they already have acquired basic competences of midwifery practice.

To what extent is it important that the following competences are developed or deepened in the Master's programme?

	1 Not important in the Master's programme [Competences sufficient through previous qualification]	2 Moderately important in the Master's programme	3 Rather important in the Master's programme	4 Important in the Master's programme	5 Very important in the Master's programme
III-6-1 Ability to use a variety of communication methods in different settings	☐	☐	☐	☐	☐
III-6-2 Ability to advocate change to improve midwifery practice and education	☐	☐	☐	☐	☐
III-6-3 Ability to apply advocacy strategies to midwifery practice and education	☐	☐	☐	☐	☐
III-6-4 Ability to act as a leader in midwifery practice and education	☐	☐	☐	☐	☐

Section X: Midwifery Educator competences: Ability to incorporate research culture into teaching

Section III: Midwifery Educator competences

X1. When students start the Master's programme, they already have acquired basic competences of midwifery practice.

To what extent is it important that the following competences are developed or deepened in the Master's programme?

	1 Not important in the Master's programme [Competences sufficient through previous qualification]	2 Moderately important in the Master's programme	3 Rather important in the Master's programme	4 Important in the Master's programme	5 Very important in the Master's programme
III-7-1 Ability to use research findings to inform teaching and practice	☐	☐	☐	☐	☐
III-7-2 Ability to promote a supportive culture for evidence-based practice	☐	☐	☐	☐	☐
III-7-3 Ability to promote a culture of critical inquiry	☐	☐	☐	☐	☐

LimeSurvey

Thank you for participating in the survey. You can now close the browser window.
