# Supplementary material for: Competency Goals in Midwifery Master’s Programs in Germany and Selected OECD Countries: Comparison of Stakeholder Perspectives
Source: Healthcare (Basel). 2026 May 18;14(10):1377. doi: 10.3390/healthcare14101377 (PMC13206547; doi:10.3390/healthcare14101377)
Supplement: Supplementary file 1 [file healthcare-14-01377-s001.zip › S4-Survey_Instrument_German.pdf]

Teil A: Persönliche Identifikationsnummer

A1.

Bitte erstellen Sie nun eine persönliche Identifikationsnummer. Diese dient einerseits der Erkennung des betroffenen Datensatzes, falls Sie Ihre Rechte, wie bereits beschrieben, auf Widerspruch oder Löschung des Datensatzes in Anspruch nehmen. Andererseits dient diese dazu, zu erkennen, ob Teilnehmende ausversehen zweimal an der Befragung teilgenommen haben. Ein Rückschluss auf Ihre Person ist nicht möglich. Bitte bewahren Sie die persönliche Identifikationsnummer an einem sicheren Platz auf. Bitte wandeln Sie Umlaute um (z.B. ö=oe) und verwenden Sie den ersten Buchstaben. Bei Nichtkenntnis tragen Sie bitte ein X ein. Beispiel: Köln, Bernd, Dezember, 16.09.1996 □KBDZ16

Die persönliche Identifikationsnummer setzt sich zusammen aus:

1. Dem ersten Buchstaben Ihres Geburtsortes

A2. **2. Dem ersten Buchstaben des Vornamens Ihres Vaters**

A3. **3. Dem letzten Buchstaben des Vornamens Ihres Vaters**

A4. **4. Dem dritten Buchstaben Ihres Geburtsmonats**

A5. **5. Den ersten zwei Ziffern Ihres Geburtstages (TT.MM.JJJJ)**

Teil B: Soziodemographische Charakteristika

B1. **Welchem Geschlecht gehören Sie an?**

- Weiblich ☐
- Männlich ☐
- Divers ☐

B2. **Wie alt sind Sie (in Jahren)?**

B3. **In welchem Land arbeiten oder studieren Sie derzeit?**

B4. **Was ist Ihr beruflicher Abschluss?**

- Abgeschlossene Berufsausbildung als Hebamme ☐
- Abgeschlossene Berufsausbildung in der Krankenpflege ☐

LimeSurvey

Abgeschlossenes Bachelorstudium (oder Gleichwertiges) in der Hebammenwissenschaft ☐

Abgeschlossenes Masterstudium (oder Gleichwertiges) in der Hebammenwissenschaft ☐

Abgeschlossenes Bachelorstudium (oder Gleichwertiges) in der Pflegewissenschaft ☐

Abgeschlossenes Masterstudium (oder Gleichwertiges) in der Pflegewissenschaft ☐

Abgeschlossenes Bachelorstudium (oder Gleichwertiges) in einer verwandten Profession ☐

Abgeschlossenes Masterstudium (oder Gleichwertiges) in einer verwandten Profession ☐

Promotion ☐

Habilitation ☐

Anderen beruflichen Abschluss ☐

B5. Haben Ihre Eltern (mindestens ein Elternteil) eine höhere Hochschulbildung abgeschlossen? (Bachelorabschluss / Äquivalent oder höher)

Ja ☐

Nein ☐

B6. Welche der folgenden Bezeichnungen trifft auf Sie zu?

Studierende/-r im Masterstudium (oder Gleichwertiges) in der Hebammenwissenschaft ☐

Dozierende/-r im Masterstudium (oder Gleichwertiges) in der Hebammenwissenschaft ☐

Arbeit in einem beruflichen Umfeld außerhalb des Hochschulbereichs mit Bezug zu Hebammenwissenschaft ☐

Dozierende/-r im Bachelorstudium (oder Gleichwertiges) in der Hebammenwissenschaft ☐

Teil C: Allgemeine Kompetenzen: Fähigkeit zur Förderung einer evidenzbasierten Praxis

Teilbereich I: Allgemeine Kompetenzen

C1. Wenn Studierende mit dem Masterstudiengang beginnen, bringen sie bereits grundlegende Kompetenzen zur Befähigung zur Hebamme mit.

In welchem Maße ist es wichtig, dass folgende Kompetenzen zusätzlich oder vertiefend im Masterstudiengang ausgebildet werden?

	1 im Masterstudiengang nicht wichtig [Kompetenzen durch Vorqualifikation ausreichend]	2 im Masterstudiengang im geringen Maße wichtig	3 im Masterst udiengang eher wichtig	4 im Masterst udiengang wichtig	5 im Masterst udiengang sehr wichtig
I-1-1 Fähigkeit, Lücken zwischen Evidenz und Praxis zu identifizieren	☐	☐	☐	☐	☐
I-1-2 Fähigkeit, Lösungen zur Überbrückung zwischen Evidenz und Praxis zu finden	☐	☐	☐	☐	☐
I-1-3 Fähigkeit, Forschungsergebnisse aus der Hebammenwissenschaft und Bezugswissenschaften in evidenzbasierte Entscheidungsprozesse zu integrieren	☐	☐	☐	☐	☐
I-1-4 Fähigkeit, evidenzbasierte Hebammenpraxis gezielt zu fördern	☐	☐	☐	☐	☐

	1 im Masterstudiengang nicht wichtig [Kompetenzen durch Vorqualifikation ausreichend]	2 im Masterstudiengang im geringen Maße wichtig	3 im Masterstudiengang eher wichtig	4 im Masterstudiengang wichtig	5 im Masterstudiengang sehr wichtig
I-1-5 Fähigkeit zur Bewertung und Nutzung von Forschungsergebnissen, um eine evidenzbasierte Gesundheitsversorgung zu ermöglichen	☐	☐	☐	☐	☐

Teil D: Allgemeine Kompetenzen: Fähigkeit zur Initiation und Koordination von Forschung im Hebammenwesen und Bewertung und Anwendung von Best-Practice-Modellen

Teilbereich I: Allgemeine Kompetenzen

D1. Wenn Studierende mit dem Masterstudiengang beginnen, bringen sie bereits grundlegende Kompetenzen zur Befähigung zur Hebamme mit.

In welchem Maße ist es wichtig, dass folgende Kompetenzen zusätzlich oder vertiefend im Masterstudiengang ausgebildet werden?

	1 im Masterstudiengang nicht wichtig [Kompetenzen durch Vorqualifikation ausreichend]	2 im Masterstudiengang im geringen Maße wichtig	3 im Masterstudiengang eher wichtig	4 im Masterstudiengang wichtig	5 im Masterstudiengang sehr wichtig
I-2-1 Fähigkeit zur Bewertung und Nutzung von Forschungsergebnissen, um Verbesserungen in der Hebammenpraxis zu initiieren	☐	☐	☐	☐	☐
I-2-2 Fähigkeit, Forschung zu initiieren und koordinieren, um die Versorgungsqualität voranzutreiben	☐	☐	☐	☐	☐
I-2-3 Fähigkeit, durch Forschungsergebnisse aus der Hebammenwissenschaft und Bezugswissenschaften, Versorgungskonzepte und -modelle zu entwickeln	☐	☐	☐	☐	☐

Teil E: Allgemeine Kompetenzen: Fähigkeit zur Initiation und Koordination von Forschung im Hebammenwesen und Bewertung und Anwendung von Best-Practice-Modellen

Teilbereich I: Allgemeine Kompetenzen

E1. Wenn Studierende mit dem Masterstudiengang beginnen, bringen sie bereits grundlegende Kompetenzen zur Befähigung zur Hebamme mit.

In welchem Maße ist es wichtig, dass folgende Kompetenzen zusätzlich oder vertiefend im Masterstudiengang ausgebildet werden?

	1 im Masterstudiengang nicht wichtig [Kompetenzen durch Vorqualifikation ausreichend]	2 im Masterstudiengang im geringen Maße wichtig	3 im Masterstudiengang eher wichtig	4 im Masterstudiengang wichtig	5 im Masterstudiengang sehr wichtig
I-2-4 Fähigkeit, klinisches Fachwissen in Best-Practice-Modellen zu bewerten und anzuwenden	☐	☐	☐	☐	☐
I-2-5 Fähigkeit, relevante Daten zur Analyse von Outcomes in der Hebammentätigkeit zu verwenden	☐	☐	☐	☐	☐

LimeSurvey

	1 im Masterstudiengang nicht wichtig [Kompetenzen durch Vorqualifikation ausreichend]	2 im Masterstudiengang im geringen Maße wichtig	3 im Masterstudiengang eher wichtig	4 im Masterstudiengang wichtig	5 im Masterstudiengang sehr wichtig
I-2-6 Fähigkeit, relevante Daten mit Best-Practice Modellen zu verknüpfen	☐	☐	☐	☐	☐

Teil F: Allgemeine Kompetenzen: Fähigkeit zur Analyse von Gesundheitspolitik

Teilbereich I: Allgemeine Kompetenzen

F1. **Wenn Studierende mit dem Masterstudiengang beginnen, bringen sie bereits grundlegende Kompetenzen zur Befähigung zur Hebamme mit.**

In welchem Maße ist es wichtig, dass folgende Kompetenzen zusätzlich oder vertiefend im Masterstudiengang ausgebildet werden?

	1 im Masterstudiengang nicht wichtig [Kompetenzen durch Vorqualifikation ausreichend]	2 im Masterstudiengang im geringen Maße wichtig	3 im Masterstudiengang eher wichtig	4 im Masterstudiengang wichtig	5 im Masterstudiengang sehr wichtig
I-3-1 Fähigkeit, Prozesse zur Entwicklung der Gesundheitspolitik zu analysieren	☐	☐	☐	☐	☐
I-3-2 Fähigkeit, einflussreiche Faktoren auf die Gesundheitspolitik zu analysieren	☐	☐	☐	☐	☐
I-3-3 Fähigkeit, Auswirkungen der Gesundheitspolitik auf die klinische Praxis zu analysieren	☐	☐	☐	☐	☐

Teil G: Allgemeine Kompetenzen: Fähigkeit zur interprofessionellen Zusammenarbeit

Teilbereich I: Allgemeine Kompetenzen

G1. **Wenn Studierende mit dem Masterstudiengang beginnen, bringen sie bereits grundlegende Kompetenzen zur Befähigung zur Hebamme mit.**

In welchem Maße ist es wichtig, dass folgende Kompetenzen zusätzlich oder vertiefend im Masterstudiengang ausgebildet werden?

	1 im Masterstudiengang nicht wichtig [Kompetenzen durch Vorqualifikation ausreichend]	2 im Masterstudiengang im geringen Maße wichtig	3 im Masterstudiengang eher wichtig	4 im Masterstudiengang wichtig	5 im Masterstudiengang sehr wichtig
I-4-1 Fähigkeit, als effektives Teammitglied in der interprofessionellen Zusammenarbeit zu agieren	☐	☐	☐	☐	☐
I-4-2 Fähigkeit, die interprofessionelle Zusammenarbeit zu verbessern, um die Gesundheitsversorgung zu fördern	☐	☐	☐	☐	☐

Teil H: Allgemeine Kompetenzen: Fähigkeit zur Identifikation von Hebammentheorien

Teilbereich I: Allgemeine Kompetenzen

H1. Wenn Studierende mit dem Masterstudiengang beginnen, bringen sie bereits grundlegende Kompetenzen zur Befähigung zur Hebamme mit.

In welchem Maße ist es wichtig, dass folgende Kompetenzen zusätzlich oder vertiefend im Masterstudiengang ausgebildet werden?

	1 im Masterstudiengang nicht wichtig [Kompetenzen durch Vorqualifikation ausreichend]	2 im Masterstudiengang im geringen Maße wichtig	3 im Masterstudiengang eher wichtig	4 im Masterstudiengang wichtig	5 im Masterstudiengang sehr wichtig
I-5-1 Fähigkeit, Theorien zu identifizieren, die für die Hebammenpraxis relevant sind	☐	☐	☐	☐	☐
I-5-2 Fähigkeit, Theorien zu identifizieren, die für die Hebammenwissenschaft relevant sind	☐	☐	☐	☐	☐
I-5-3 Fähigkeit, Theorien zu identifizieren, die für die Förderung von gesundheitlicher Chancengleichheit und sozialer Gerechtigkeit relevant sind	☐	☐	☐	☐	☐
I-5-4 Fähigkeit, Theorien zu identifizieren, die für ethische Grundsätze in der Hebammentätigkeit relevant sind	☐	☐	☐	☐	☐

Teil I: Allgemeine Kompetenzen: Fähigkeit zur Technologienutzung

Teilbereich I: Allgemeine Kompetenzen

I1. Wenn Studierende mit dem Masterstudiengang beginnen, bringen sie bereits grundlegende Kompetenzen zur Befähigung zur Hebamme mit.

In welchem Maße ist es wichtig, dass folgende Kompetenzen zusätzlich oder vertiefend im Masterstudiengang ausgebildet werden?

	1 im Masterstudiengang nicht wichtig [Kompetenzen durch Vorqualifikation ausreichend]	2 im Masterstudiengang im geringen Maße wichtig	3 im Masterstudiengang eher wichtig	4 im Masterstudiengang wichtig	5 im Masterstudiengang sehr wichtig
I-6-1 Fähigkeit, Technologien zu nutzen, um die Qualität der Gesundheitsversorgung zu verbessern	☐	☐	☐	☐	☐
I-6-2 Fähigkeit, Technologien zu nutzen, um die Sicherheit der Gesundheitsversorgung zu verbessern	☐	☐	☐	☐	☐

Teil J: Allgemeine Kompetenzen: Fähigkeit zum Management von Finanzen und Ressourcen

Teilbereich I: Allgemeine Kompetenzen

J1. **Wenn Studierende mit dem Masterstudiengang beginnen, bringen sie bereits grundlegende Kompetenzen zur Befähigung zur Hebamme mit.**

In welchem Maße ist es wichtig, dass folgende Kompetenzen zusätzlich oder vertiefend im Masterstudiengang ausgebildet werden?

	1 im Masterstudiengang nicht wichtig [Kompetenzen durch Vorqualifikation ausreichend]	2 im Masterstudiengang im geringen Maße wichtig	3 im Masterstudiengang eher wichtig	4 im Masterstudiengang wichtig	5 im Masterstudiengang sehr wichtig
I-7-1 Fähigkeit, finanzielle Aspekte des Gesundheitswesens zu bewerten	☐	☐	☐	☐	☐
I-7-2 Fähigkeit, Ressourcen angemessen im Gesundheitswesen einzusetzen	☐	☐	☐	☐	☐
I-7-3 Fähigkeit, die Ressourcen einer Praxis im Gesundheitswesen zu managen	☐	☐	☐	☐	☐

Teil K: Allgemeine Kompetenzen: Fähigkeit zur Berücksichtigung des professionellen Berufsverständnisses der Hebammen in verschiedenen Kontexten

Teilbereich I: Allgemeine Kompetenzen

K1. **Wenn Studierende mit dem Masterstudiengang beginnen, bringen sie bereits grundlegende Kompetenzen zur Befähigung zur Hebamme mit.**

In welchem Maße ist es wichtig, dass folgende Kompetenzen zusätzlich oder vertiefend im Masterstudiengang ausgebildet werden?

	1 im Masterstudiengang nicht wichtig [Kompetenzen durch Vorqualifikation ausreichend]	2 im Masterstudiengang im geringen Maße wichtig	3 im Masterstudiengang eher wichtig	4 im Masterstudiengang wichtig	5 im Masterstudiengang sehr wichtig
I-8-1 Fähigkeit, das professionelle Berufsverständnis der Hebammen in der klinischen Praxis zu berücksichtigen	☐	☐	☐	☐	☐
I-8-2 Fähigkeit, das professionelle Berufsverständnis der Hebammen in der Verwaltung zu berücksichtigen	☐	☐	☐	☐	☐
I-8-3 Fähigkeit, das professionelle Berufsverständnis der Hebammen bei der Umsetzung politischer Maßnahmen zu berücksichtigen	☐	☐	☐	☐	☐
I-8-4 Fähigkeit, gesundheitliche Ungleichheit in der Hebammentätigkeit zu berücksichtigen	☐	☐	☐	☐	☐
I-8-5 Fähigkeit, potenzielle Interessensgebiete der Hebammenwissenschaft zu erkunden	☐	☐	☐	☐	☐

Teil L: Grundlegende Fähigkeiten

Teilbereich I: Allgemeine Kompetenzen

L1. Wenn Studierende mit dem Masterstudiengang beginnen, bringen sie bereits grundlegende Kompetenzen zur Befähigung zur Hebamme mit.

In welchem Maße ist es wichtig, dass folgende Kompetenzen zusätzlich oder vertiefend im Masterstudiengang ausgebildet werden?

	1 im Masterstudiengang nicht wichtig [Kompetenzen durch Vorqualifikation ausreichend]	2 im Masterstudiengang im geringen Maße wichtig	3 im Masterstudiengang eher wichtig	4 im Masterstudiengang wichtig	5 im Masterstudiengang sehr wichtig
C-1 Fähigkeit, grundlegende Menschenrechte im Rahmen der Hebammentätigkeit einzuhalten	☐	☐	☐	☐	☐
C-2 Fähigkeit, gesetzliche Vorschriften, Regulierungsauflagen und Verhaltenskodizes in Bezug auf die Hebammentätigkeit einzuhalten	☐	☐	☐	☐	☐
C-3 Fähigkeit, die normalen Geburtsprozesse in Institutionen und im außerklinischen Bereich zu fördern	☐	☐	☐	☐	☐
C-4 Fähigkeit, Normabweichungen und Komplikationen zu erkennen und eine angemessene Behandlung oder Überweisung einzuleiten	☐	☐	☐	☐	☐

Teil M: Advanced Midwifery Practice Kompetenzen: Fähigkeit zur Gewährleistung der Würde, Vertrauen und Diskretion

Teilbereich II: Advanced Midwifery Practice Kompetenzen

M1. Wenn Studierende mit dem Masterstudiengang beginnen, bringen sie bereits grundlegende Kompetenzen zur Befähigung zur Hebamme mit.

In welchem Maße ist es wichtig, dass folgende Kompetenzen zusätzlich oder vertiefend im Masterstudiengang ausgebildet werden?

	1 im Masterstudiengang nicht wichtig [Kompetenzen durch Vorqualifikation ausreichend]	2 im Masterstudiengang im geringen Maße wichtig	3 im Masterstudiengang eher wichtig	4 im Masterstudiengang wichtig	5 im Masterstudiengang sehr wichtig
II-1-1 Fähigkeit, ethisch fundierte Lösungen für komplexe Fragen im Zusammenhang mit der Betreuung von Frauen und ihren Babys anzuwenden	☐	☐	☐	☐	☐
II-1-2 Fähigkeit, die Rechte und Wünsche von Frauen, ihren Babys und Familien bei anderen Gesundheitsfachkräften und Gesundheitsdienstleistungen zu verhandeln und zu vertreten	☐	☐	☐	☐	☐

LimeSurvey

Teil N: Advanced Midwifery Practice Kompetenzen: Fähigkeit zur Übernahme von beruflicher Verantwortung und Rechenschaftspflicht in leitender Funktion

Teilbereich II: Advanced Midwifery Practice Kompetenzen

N1. Wenn Studierende mit dem Masterstudiengang beginnen, bringen sie bereits grundlegende Kompetenzen zur Befähigung zur Hebamme mit.

In welchem Maße ist es wichtig, dass folgende Kompetenzen zusätzlich oder vertiefend im Masterstudiengang ausgebildet werden?

	1 im Masterstudiengang nicht wichtig [Kompetenzen durch Vorqualifikation ausreichend]	2 im Masterstudiengang im geringen Maße wichtig	3 im Masterstudiengang eher wichtig	4 im Masterstudiengang wichtig	5 im Masterstudiengang sehr wichtig
II-2-1 Fortgeschrittene Kenntnisse, Fähigkeiten und Fertigkeiten einsetzen, um in leitender Funktion komplexe klinische Entscheidungen zu treffen	☐	☐	☐	☐	☐
II-2-2 Fortgeschrittene Kenntnisse, Fähigkeiten und Fertigkeiten einsetzen, um in leitender Position Probleme kritisch zu analysieren und zu lösen	☐	☐	☐	☐	☐

Teil O: Advanced Midwifery Practice Kompetenzen: Fähigkeit zum Schutz und zur Förderung der Qualität der Praxis

Teilbereich II: Advanced Midwifery Practice Kompetenzen

O1. Wenn Studierende mit dem Masterstudiengang beginnen, bringen sie bereits grundlegende Kompetenzen zur Befähigung zur Hebamme mit.

In welchem Maße ist es wichtig, dass folgende Kompetenzen zusätzlich oder vertiefend im Masterstudiengang ausgebildet werden?

	1 im Masterstudiengang nicht wichtig [Kompetenzen durch Vorqualifikation ausreichend]	2 im Masterstudiengang im geringen Maße wichtig	3 im Masterstudiengang eher wichtig	4 im Masterstudiengang wichtig	5 im Masterstudiengang sehr wichtig
II-3-1 Fähigkeit, qualitativ hochwertige Mutterschaftsbetreuung zu fördern und zu schützen	☐	☐	☐	☐	☐
II-3-2 Fähigkeit, mitfühlende Mutterschaftsbetreuung zu fördern und zu schützen	☐	☐	☐	☐	☐
II-3-3 Fähigkeit, evidenzbasierte sichere Mutterschaftsbetreuung zu fördern und zu schützen	☐	☐	☐	☐	☐

Teil P: Advanced Midwifery Practice Kompetenzen: Fähigkeit zur gemeinsamen Risikobewertung

Teilbereich II: Advanced Midwifery Practice Kompetenzen

P1. Wenn Studierende mit dem Masterstudiengang beginnen, bringen sie bereits grundlegende Kompetenzen zur Befähigung zur Hebamme mit.

In welchem Maße ist es wichtig, dass folgende Kompetenzen zusätzlich oder vertiefend im Masterstudiengang ausgebildet werden?

	1 im Masterstudiengang nicht wichtig [Kompetenzen durch Vorqualifikation ausreichend]	2 im Masterstudiengang im geringen Maße wichtig	3 im Masterstudiengang eher wichtig	4 im Masterstudiengang wichtig	5 im Masterstudiengang sehr wichtig
II-4-1 Fähigkeit, eine gemeinsame Risikobewertung in der Zusammenarbeit mit anderen zu steuern	☐	☐	☐	☐	☐
II-4-2 Fähigkeit, eine sichere Umgebung in der Zusammenarbeit mit anderen zu fördern	☐	☐	☐	☐	☐

Teil Q: Hebammen Lehrende/-r Kompetenzen: Fähigkeit zum Einbezug von ethischen und rechtlichen Grundsätzen in der Lehre

Teilbereich III: Hebammen Lehrende/-r Kompetenzen

Q1. Wenn Studierende mit dem Masterstudiengang beginnen, bringen sie bereits grundlegende Kompetenzen zur Befähigung zur Hebamme mit.

In welchem Maße ist es wichtig, dass folgende Kompetenzen zusätzlich oder vertiefend im Masterstudiengang ausgebildet werden?

	1 im Masterstudiengang nicht wichtig [Kompetenzen durch Vorqualifikation ausreichend]	2 im Masterstudiengang im geringen Maße wichtig	3 im Masterstudiengang eher wichtig	4 im Masterstudiengang wichtig	5 im Masterstudiengang sehr wichtig
III-1-1 Fähigkeit, ethische Aspekte der Hebammentätigkeit in die Lehr/-Lernaktivität einzubeziehen	☐	☐	☐	☐	☐
III-1-2 Fähigkeit, rechtliche Aspekte der Hebammentätigkeit in die Lehr/-Lernaktivität einzubeziehen	☐	☐	☐	☐	☐
III-1-3 Fähigkeit, konsequentes Vorbildverhalten bzgl. ethischer und rechtlicher Aspekte in der Lehr/-Lernaktivität zu fördern	☐	☐	☐	☐	☐

LimeSurvey

Teil R: Hebammen Lehrende/-r Kompetenzen: Fähigkeit zur Aufrechterhaltung und Aktualisierung von Kompetenzen in Theorie und Praxis

Teilbereich III: Hebammen Lehrende/-r Kompetenzen

R1. **Wenn Studierende mit dem Masterstudiengang beginnen, bringen sie bereits grundlegende Kompetenzen zur Befähigung zur Hebamme mit.**

In welchem Maße ist es wichtig, dass folgende Kompetenzen zusätzlich oder vertiefend im Masterstudiengang ausgebildet werden?

	1 im Masterstudiengang nicht wichtig [Kompetenzen durch Vorqualifikation ausreichend]	2 im Masterstudiengang im geringen Maße wichtig	3 im Masterstudiengang eher wichtig	4 im Masterstudiengang wichtig	5 im Masterstudiengang sehr wichtig
III-2-1 Fähigkeit, Kenntnisse und Fähigkeiten in Theorie und Praxis der Hebammentätigkeit auf dem neusten Stand zu halten	☐	☐	☐	☐	☐
III-2-2 Fähigkeit, Kenntnisse und Fähigkeiten in Theorie und Praxis der Hebammentätigkeit auf die beste verfügbare Evidenz zu stützen	☐	☐	☐	☐	☐

Teil S: Hebammen Lehrende/-r Kompetenzen: Fähigkeit zur Schaffung eines lernförderlichen Umfelds für theoretisches Lernen

Teilbereich III: Hebammen Lehrende/-r Kompetenzen

S1. **Wenn Studierende mit dem Masterstudiengang beginnen, bringen sie bereits grundlegende Kompetenzen zur Befähigung zur Hebamme mit.**

In welchem Maße ist es wichtig, dass folgende Kompetenzen zusätzlich oder vertiefend im Masterstudiengang ausgebildet werden?

	1 im Masterstudiengang nicht wichtig [Kompetenzen durch Vorqualifikation ausreichend]	2 im Masterstudiengang im geringen Maße wichtig	3 im Masterstudiengang eher wichtig	4 im Masterstudiengang wichtig	5 im Masterstudiengang sehr wichtig
III-3-1 Fähigkeit, pädagogische Strategien zur Förderung des aktiven Lernens einzubeziehen	☐	☐	☐	☐	☐
III-3-2 Fähigkeit, effektive Lehr- und Lernmaterialien auszuwählen und einzusetzen	☐	☐	☐	☐	☐
III-3-3 Fähigkeit, unterschiedliche Lernstile und individuelle Lernbedürfnisse zu erkennen und zu unterstützen	☐	☐	☐	☐	☐

LimeSurvey

Teil T: Hebammen Lehrende/-r Kompetenzen: Fähigkeit zur Schaffung lernförderlichen Umfelds für klinische Lehre

Teilbereich III: Hebammen Lehrende/-r Kompetenzen

T1. Wenn Studierende mit dem Masterstudiengang beginnen, bringen sie bereits grundlegende Kompetenzen zur Befähigung zur Hebamme mit.

In welchem Maße ist es wichtig, dass folgende Kompetenzen zusätzlich oder vertiefend im Masterstudiengang ausgebildet werden?

	1 im Masterstudiengang nicht wichtig [Kompetenzen durch Vorqualifikation ausreichend]	2 im Masterstudiengang im geringen Maße wichtig	3 im Masterstudiengang eher wichtig	4 im Masterstudiengang wichtig	5 im Masterstudiengang sehr wichtig
III-4-1 Fähigkeit, ein sicheres und effektives Lernumfeld in der klinischen Umgebung zu schaffen	☐	☐	☐	☐	☐
III-4-2 Fähigkeit, individuelles Erfahrungslernen zu fördern	☐	☐	☐	☐	☐

Teil U: Hebammen Lehrende/-r Kompetenzen: Fähigkeit zur Qualitätssicherung in Bildungsprogrammen

Teilbereich III: Hebammen Lehrende/-r Kompetenzen

U1. Wenn Studierende mit dem Masterstudiengang beginnen, bringen sie bereits grundlegende Kompetenzen zur Befähigung zur Hebamme mit.

In welchem Maße ist es wichtig, dass folgende Kompetenzen zusätzlich oder vertiefend im Masterstudiengang ausgebildet werden?

	1 im Masterstudiengang nicht wichtig [Kompetenzen durch Vorqualifikation ausreichend]	2 im Masterstudiengang im geringen Maße wichtig	3 im Masterstudiengang eher wichtig	4 im Masterstudiengang wichtig	5 im Masterstudiengang sehr wichtig
III-5-1 Fähigkeit, das Bildungsprogramm für Hebammen regelmäßig zu überwachen, zu bewerten und zu evaluieren	☐	☐	☐	☐	☐
III-5-2 Fähigkeit, die Kompetenzen der Studierenden regelmäßig zu bewerten	☐	☐	☐	☐	☐

LimeSurvey

Teil V: Hebammen Lehrende/-r Kompetenzen: Fähigkeit zur Qualitätssicherung in Bildungsprogrammen

Teilbereich III: Hebammen Lehrende/-r Kompetenzen

V1. Wenn Studierende mit dem Masterstudiengang beginnen, bringen sie bereits grundlegende Kompetenzen zur Befähigung zur Hebamme mit.

In welchem Maße ist es wichtig, dass folgende Kompetenzen zusätzlich oder vertiefend im Masterstudiengang ausgebildet werden?

	1 im Masterstudiengang nicht wichtig [Kompetenzen durch Vorqualifikation ausreichend]	2 im Masterstudiengang im geringen Maße wichtig	3 im Masterstudiengang eher wichtig	4 im Masterstudiengang wichtig	5 im Masterstudiengang sehr wichtig
III-5-3 Fähigkeit, sich aktiv an der Organisation und Umsetzung eines Curriculums für die Hebammenwissenschaft zu beteiligen	☐	☐	☐	☐	☐
III-5-4 Fähigkeit, Bildungsprogramme für Hebammen zu überarbeiten und umzusetzen	☐	☐	☐	☐	☐

Teil W: Hebammen Lehrende/-r Kompetenzen: Fähigkeit zur Interessenvertretung und als Führungskraft zu agieren

Teilbereich III: Hebammen Lehrende/-r Kompetenzen

W1. Wenn Studierende mit dem Masterstudiengang beginnen, bringen sie bereits grundlegende Kompetenzen zur Befähigung zur Hebamme mit.

In welchem Maße ist es wichtig, dass folgende Kompetenzen zusätzlich oder vertiefend im Masterstudiengang ausgebildet werden?

	1 im Masterstudiengang nicht wichtig [Kompetenzen durch Vorqualifikation ausreichend]	2 im Masterstudiengang im geringen Maße wichtig	3 im Masterstudiengang eher wichtig	4 im Masterstudiengang wichtig	5 im Masterstudiengang sehr wichtig
III-6-1 Fähigkeit, eine Vielzahl von Kommunikationsmethoden in unterschiedlichen Umgebungen einzusetzen	☐	☐	☐	☐	☐
III-6-2 Fähigkeit, sich für Veränderungen zur Verbesserung der Hebammenpraxis und dem Hebammenstudium einzusetzen	☐	☐	☐	☐	☐
III-6-3 Fähigkeit, Interessensvertretungsstrategien für die Hebammenpraxis und dem Hebammenstudium anzuwenden	☐	☐	☐	☐	☐
III-6-4 Fähigkeit, als Führungskraft in der Hebammenpraxis und dem Hebammenstudium zu agieren	☐	☐	☐	☐	☐

Teil X: Hebammen Lehrende/-r Kompetenzen: Fähigkeit zur Berücksichtigung von Forschungskultur in der Lehre

Teilbereich III: Hebammen Lehrende/-r Kompetenzen

X1. Wenn Studierende mit dem Masterstudiengang beginnen, bringen sie bereits grundlegende Kompetenzen zur Befähigung zur Hebamme mit.

In welchem Maße ist es wichtig, dass folgende Kompetenzen zusätzlich oder vertiefend im Masterstudiengang ausgebildet werden?

	1 im Masterstudiengang nicht wichtig [Kompetenzen durch Vorqualifikation ausreichend]	2 im Masterstudiengang im geringen Maße wichtig	3 im Masterstudiengang eher wichtig	4 im Masterstudiengang wichtig	5 im Masterstudiengang sehr wichtig
III-7-1 Fähigkeit, Forschungsergebnisse als Grundlage für Lehre und Praxis zu nutzen	☐	☐	☐	☐	☐
III-7-2 Fähigkeit, eine Kultur zu fördern, die evidenzbasierte Praxis unterstützt	☐	☐	☐	☐	☐
III-7-3 Fähigkeit, eine Kultur zu fördern, die kritisch hinterfragt	☐	☐	☐	☐	☐

Vielen Dank für Ihre Teilnahme an der Befragung. Sie können das Browser-Fenster nun schließen.
